# Improving compliance around protected areas through fair administration of rules *

**DOI:** 10.1111/cobi.14332

**Published:** 2024-07-17

**Authors:** Harriet Ibbett, Leejiah Dorward, Julia P.G. Jones, Edward M. Kohi, Asri A. Dwiyahreni, Stephen Sankeni, Karlina Prayitno, Jesca Mchomvu, Joseph Kaduma, Andie Wijaya Saputra, Ika Yuni Agustin, Tyassanti Tryswidiarini, Rose Mawenya, Jatna Supriatna, Freya A.V. St John

**Affiliations:** 1 School of Natural Sciences, Bangor University, Bangor, UK; 2 Research Centre for Climate Change, Universitas Indonesia, Depok, Indonesia; 3 Tanzania Wildlife Research Institute (TAWIRI), Arusha, Tanzania; 4 Conservation and Human Behaviour Research Group, Bangor University, Bangor, UK

**Keywords:** conservation conflict, conservation law enforcement, corruption, deterrence theory, Indonesia, legitimacy, rule breaking, Tanzania

## Abstract

Protected area management often depends heavily on law enforcement to secure compliance with rules. However, this can contribute to conflict between protected area authorities and local people, negatively affecting both human well-being and conservation outcomes. Compliance is affected by many factors, including whether those who enforce rules are perceived to do so fairly, as well as the perceived rule-related behavior of others. We used factorial survey experiments to explore how fair respondents living around protected areas in Indonesia and Tanzania perceive sanctions distributed by law enforcers to be. We presented scenarios to respondents to assess how crime type, offender characteristics, and corruption influenced their judgments regarding the fairness of administered sanctions. We also assessed how descriptive norms and corruption influenced individuals’ willingness to obey protected area rules. Data were collected from 229 people in Indonesia and 217 in Tanzania. Results showed that in both locations, lawful sanctions, such as arrests or warnings, were perceived as fairer, and sanctions that involved corruption were perceived as least fair. Attitudes toward protected area rules, corruption, and descriptive norms all influenced people’s willingness to comply, whereas multidimensional poverty did not. Our results highlight the need for conservation policy and practice to move beyond narratives that focus on the need for more law enforcement. To improve protected area compliance and secure better outcomes for people and nature, conservation must focus on ensuring the fair administration of rules and enhancing the legitimacy of rules themselves.

## Introduction

Rules restricting and regulating access to resources are an integral component of protected area management, yet to be effective, rules must be complied with (Arias, 2015; Matseketsa et al., 2022). Law enforcement—the monitoring of adherence to rules and the punishment of detected infractions—has long been the dominant strategy for generating compliance in conservation (Keane et al., 2008). Grounded in economics, law enforcement emphasizes the role of rational choice and deterrence theory in influencing individuals’ decisions to comply, with the underlying hypothesis being that people break rules when anticipated benefits outweigh costs (Becker, 1974). Consequently, conservation authorities spend significant resource deploying law enforcers, such as rangers, park guards, the army, or police, to patrol protected areas, detect infractions, administer sanctions, and process offenders for prosecution (Massé, 2020; Moreto & Gau, 2017). In some places, such investment has also been accompanied by increased militarization of law enforcement (Ashaba, 2021; Duffy, 2014). Yet, securing compliance through coercive means can contribute to conflict between authorities and local people, undermining conservation outcomes (Verweijen & Marijnen, 2018; Witter, 2021). This may be particularly so in contexts of contested illegality where the rules themselves are seen as illegitimate (Hübschle, 2017a) or in circumstances where the legitimacy of protected areas and law enforcement authorities is already questioned, for example, where associated with colonial regimes, or practices of exclusion, dispossession, or displacement (Akampurira, 2023; Duffy et al., 2019; Hübschle, 2017b; Robbins et al., 2005). Understanding the normative factors that influence compliance, including whether those who make and enforce protected area rules are perceived as legitimate and to enforce rules and distribute sanctions fairly, is thus essential to improving outcomes for people and nature (Kahler & Gore, 2012; Stern, 2008).

Legitimate authorities are those that are recognized as proper, just, and worthy of power (Tyler, 2021). Legitimacy encourages greater voluntary compliance and is strongly affected by people’s personal experiences with authorities, including whether individuals are treated fairly and with respect and whether sanctions are administered consistently and impartially (Sunshine & Tyler, 2003). This matters because interactions between authorities and local people around protected areas often involve discretion. Officers distribute sanctions according to a range of legal and extralegal factors, including offender demographics and transgression severity (Carter, 2006; Eliason, 2003). For example, wildlife conservation officers in South Africa reported they sanctioned illegal hunters according to their age. Older perpetrators were taken to the police, whereas young offenders were taken home to be sanctioned by elders (Warchol & Kapla, 2012). However, if the decisions and sanctions imparted by authorities are perceived as inconsistent or unfair, it can undermine their legitimacy. For example, residents living around the Makira-Masoala Conservation Area in Madagascar felt it unfair that rich offenders received more privileged treatment when they broke conservation rules compared with the poor (Gore et al., 2013), and people living in Seima Wildlife Sanctuary in Cambodia similarly objected to outsiders, or those with power, being treated differently by authorities (Ibbett et al., 2021).

Legitimacy is also affected by how authorities use their power (Trinkner et al., 2018). Sometimes local people may feel authorities should exercise power beyond that allowed by law (Trinkner et al., 2018). For example, when enforcing rules in their own community, in cases where perpetrators are poor, or in contexts where communities have historically harvested natural resources to meet subsistence needs, communities may perceive authorities as more legitimate if they use their power compassionately to excercise discretion (Belecky, Moreto, et al., 2021; Trinkner et al., 2018). Indeed, sanctions levied upon people trying to sustain their livelihoods may lead people to view authorities as uncaring, arbitrary, and untrustworthy (Moreto & Gau, 2017).

Conversely, abuse of power can seriously undermine legitimacy. Many conservation law enforcers are poorly and infrequently paid, inadequately equipped, and tasked with protecting landscapes rich in high-value resources, often in remote areas with little oversight and in contexts with weak governance and high social–economic inequality, providing plentiful incentives and opportunities to misuse power for personal gain (Belecky, Moreto, et al., 2021). Corruption, defined as the use of public office for private gain (Wilson & Damania, 2005), occurs in a variety of ways, including when law enforcers collude with rule breakers (e.g., by accepting bribes), willfully neglect their duties (Moreto et al., 2015), share enforcement information (Mmahi & Usman, 2020), and break rules themselves (Moreto et al., 2015). Such behavior can become pervasive because those who collude with law enforcers are rarely incentivized to report or protest against the misuse of power (Smith et al., 2003). All these behaviors corrode trust in authorities and contribute to reduced compliance (Huq et al., 2016; Kahler et al., 2013; Levi et al., 2009; Moreto & Gau, 2017).

Additionally, other social–psychological factors, such as what others think (injunctive norms) and what others do (descriptive norms), determine desirable behavior (Cialdini & Trost, 1998) and can significantly affect the perceived legitimacy of rules (Hübschle, 2017a), which in turn can both foster and hinder compliance (Arias, 2015; Bragagnolo et al., 2017; Ramcilovic-Suominen & Epstein, 2012; St John et al., 2015). Norms affect compliance with rules governing natural resource use across multiple contexts. For example, in Nigeria, whether friends and family followed rules regarding hunting in protected areas has a significant impact on individuals’ intention to comply (Atuo et al., 2020), and norms influence individuals’ decisions to obey fishing regulations in Chile (Oyanedel et al., 2020).

A key challenge to researching topics such as conservation law enforcement, compliance, and corruption is that people are often unwilling to reveal their involvement or freely discuss their perspectives for fear of repercussions (Nuno & St John, 2015; Robbins, 2000), which affects data quality (Krumpal, 2013; Tourangeau & Yan, 2007) and participant and researcher safety (Ibbett et al., 2023). Employing scenario-based approaches provides one means of overcoming some of these barriers. Instead of asking about respondents’ behavior, factorial survey experiments (also known as vignette experiments) use hypothetical scenarios to elicit people’s preferences, behavioral intentions, and decision-making processes in different situations (Auspurg & Hinz, 2014; Rossi & Nock, 1982) and have been successfully applied to investigate sensitive topics across numerous disciplines including crime, law, and deviance (Wallander, 2009).

We used factorial survey experiments to explore how fair respondents living around protected areas in Indonesia and Tanzania perceive sanctioning by law enforcers to be. We presented respondents with vignettes that differed in a discrete number of factors and asked them to evaluate each one according to predefined criteria (Auspurg & Hinz, 2014). We specifically assessed how crime type, offender characteristics (including where an offender is from and the offender’s relative power), and corruption influenced respondents’ judgments regarding the fairness of the sanction administered. We then assessed how respondents’ willingness to comply with protected area rules was affected by corruption, descriptive norms, and demographic factors, including age, gender, and multidimensional poverty status.

## Methods

### Study sites

**Figure 1:**
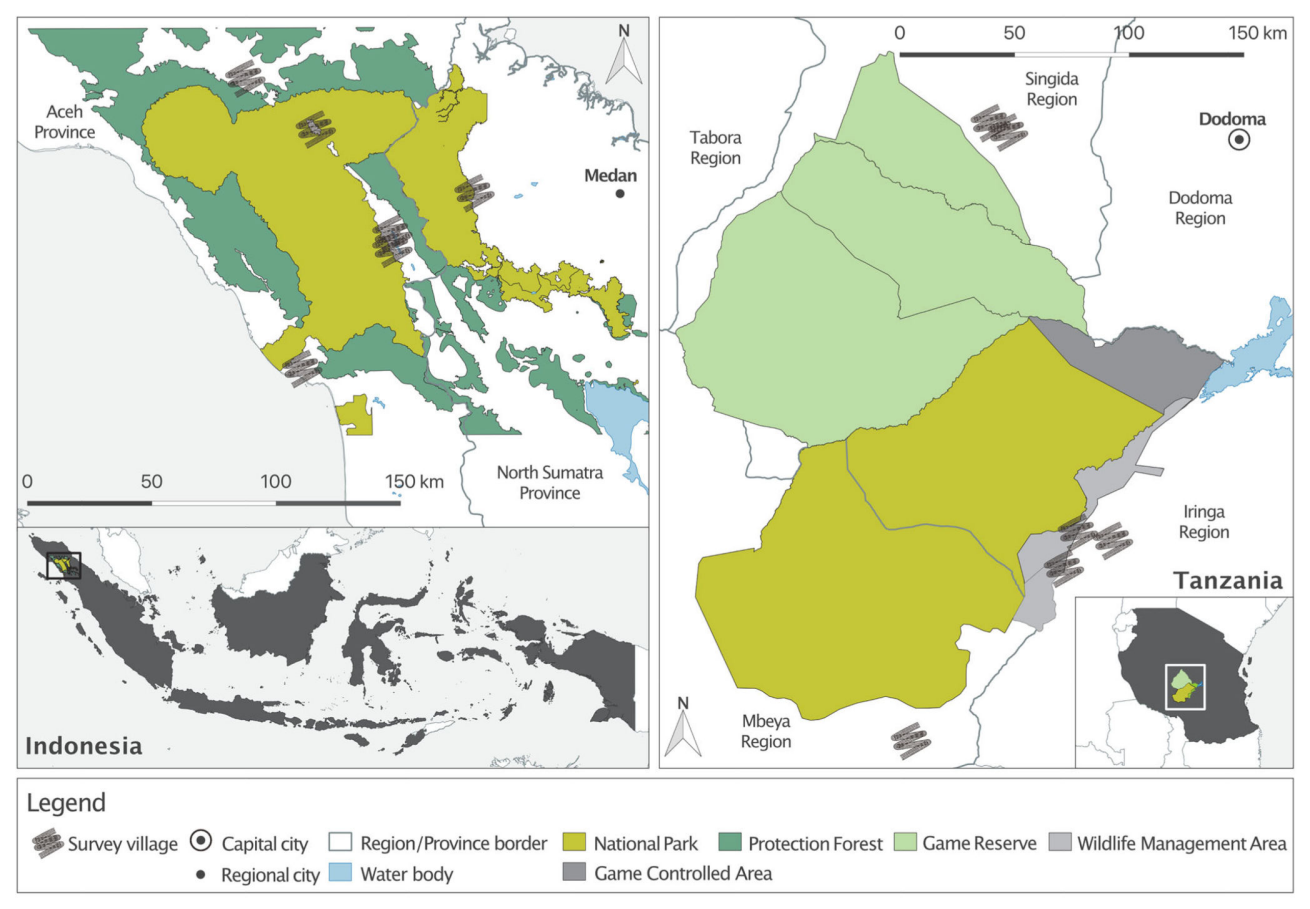
Survey locations around Gunung Leuser National Park in northern Sumatra, Indonesia (right, 6 villages), and Ruaha–Rungwa ecosystem in central Tanzania (left, 6 villages). In line with ethics approval, precise locations of study villages are not indicated.

Working in 2 countries provided a valuable opportunity to compare findings across contrasting cultural and socioeconomic contexts. We conducted research in 6 villages situated around Gunung Leuser National Park in northern Sumatra, Indonesia, and 6 villages located in the Ruaha–Rungwa ecosystem in central Tanzania (Figure 1). We selected these sites because both countries are of global importance for biodiversity (Olson & Dinerstein, 1998), rate poorly on the global corruption perception index (Indonesia: score 34, rank 110; Tanzania: score 38, rank 94; score range 0, highly corrupt, to 100, not corrupt; rank is out of 180 countries [Transparency International, 2022]), but differ in their positions on the global development trajectory. Indonesia is characterized by relatively low levels of multidimensional poverty, whereas Tanzania is not (United Nations, 2023). Both countries have extensive protected area networks established by colonial administrations associated with contested environmental histories and controversial practices, including dispossession (Boomgaard, 1999; Brockington et al., 2008), that restrict local people’s access to natural resources. Relationships between local people and protected area authorities remain understudied in both landscapes.

### Survey instrument

Recognizing that there are shortcomings associated with translating response scales into different languages and knowledge systems (Cha et al.,2007), we aimed to minimize these risks as much as possible. The survey instrument was developed with all members of the research teams, which included individuals from both study countries. Together, we developed our questionnaire in English, translated it into Indonesian and Kiswahili, and independently back translated it prior to piloting. It was administered face-to-face, with statements and response options relayed verbally. Due to the sensitive nature of the subject, village names and participants’ responses were anonymized. Responses were recorded on mobile phones and encrypted at the point of capture. Because we were interested in perceptions of people living across the landscape, we adopted a convenience sampling strategy (Newing, 2011); access to participants was facilitated with the help of village leaders. We selected villages across the landscape within 10 km of protected areas. Only individuals 18 years old or older were surveyed, with free, prior, and informed consent sought verbally from all participants. Research was approved by Bangor University Ethics Committee (coses2021hi01) and conducted with the permission of national and local authorities (COSTECH permit No. 2021-426-NA-2021-224, RISTEK permit No. 6/E5/E5.4/SIP.EXT/2021). Data were collected in Indonesia from September 2021 to February 2022 and in Tanzania from January to February 2022.

We first captured demographic characteristics, including respondent age, gender, and years of formal education completed. We incorporated questions about household education, health, and living standards to calculate a household measure of multidimensional poverty (Alkire et al., 2020) (Appendix S1).

### Perceived fairness of sanctions (Experiment 1)

To explore the perceived fairness of sanctions administered by law enforcers, we presented respondents with a series of vignettes in which an offender was caught breaking protected area rules. Vignettes varied across 4 factors (Table 1): crime committed; whether the offender was from the same community as the respondent; whether the offender held power (e.g., social, or financial standing that enables exertion of control or influence over others); and the sanction administered.

We selected these factors for several reasons. In both countries, we wanted to include crimes commonly studied within conservation (e.g., wildlife hunting) and identified as being of specific concern within the study sites. Previous research suggests that hunting wildlife (assessed in both countries), grazing livestock (Tanzania only), and logging high-value timber (Indonesia only) were conducted by people living in surrounding villages, as well as people from elsewhere. In Indonesia, logging within the protected area was considered more sensitive than hunting protected wildlife such as sambar, whereas in Tanzania, hunting wildlife and grazing livestock were considered equally sensitive (Ibbett et al., 2023). In both contexts, during preliminary research, we encountered reports of outsiders exerting power or receiving preferential treatment during interactions with law enforcers, which we wanted to explore further. To assess how rangers’ use of authority influenced perceived fairness, 2 lawful sanctions were included (arrest and prosecution and formal warning with resources confiscated) alongside law enforcers’ use of discretion (no sanction) and abuse of power (bribery). Respondents were asked to evaluate each vignette reporting how fair they perceived the sanction to be on a 5-point Likert scale.

Following Lawson et al. (2009), we generated a mixed-level (one factor had 4 levels and 3 factors had 2 levels [4 × 2 × 2 × 2]) full factorial design (meaning every possible combination of factors and levels was included) composed of 32 vignettes. The vignettes were divided into 4 blocks of 8 with respondents randomly allocated to one block, and the order vignettes were presented to respondents was randomized to minimize order effects (Appendix S1). The design was orthogonal and balanced, with a *D* efficiency of 100, meaning all main effects and interactions could be estimated independently (Dülmer, 2016; Kleinewiese, 2022). The *D* efficiency identifies the precision with which parameters can be estimated (100, maximum efficiency).

### Impact of corruption and descriptive norms on willingness to comply (Experiment 2)

To explore how corruption and descriptive norms affected an individual’s willingness to follow protected area rules, we first measured respondents’ attitude toward protected area rules by assessing agreement with the following statement: “The rules of the protected area are fair and consistent with the law.” We then randomly allocated respondents to one of 4 vignettes and asked given the scenario “How willing would you be to follow protected area rules?” Answers were reported using a 5-point Likert scale (from *very willing* to *very unwilling*). The scenarios contained 2 factors, each with 2 levels: corruption (whether law enforcers were likely to accept a bribe or not) and descriptive norms (whether compliance of others in the community was high or low) (Table 2). This design is an adaptation of Sundström (2016).

### Data analyses

In Experiment 1, perceived fairness is modeled as an ordinal response variable with the R package ordinal (Christensen, 2019; R Core Team, 2023). The distribution of our data was strongly polarized across the response options; respondents rarely used the middle agree−disagree options. Thus, to improve model interpretability, we condensed our 5-point scale to a 3-point scale (Agree, Neutral, Disagree). Although this can result in loss of information, it is an approach commonly employed to overcome this challenge (Allen & Seaman, 2007). We included the 4 vignette factors (crime committed, where the offender was from, the power of the offender, and sanction administered) as predictors alongside interactions between the sanction administered and each other factor. Because data were grouped, with each respondent answering multiple vignettes, we included individual respondent identity as a random effect. We included only respondents who answered all 8 vignettes, and to minimize bias, we excluded all cases where respondents provided the same response across all vignettes (Auspurg & Hinz, 2014). Because the questionnaire design differed between countries (i.e., we used different levels for the crime committed factor) and we wished to be able to make country-specific statements about the findings, we ran separate models for each country. Data were collected from 229 people in Indonesia and 217 in Tanzania. Eight respondents in Indonesia and 3 in Tanzania did not evaluate all 8 vignettes in Experiment 1 and were excluded from analysis. Vignette responses from a further 5 individuals in Indonesia and 6 in Tanzania were also discarded because they showed no variation in the response pattern, suggesting low engagement or potential acquiesce bias.

We conducted similar analyses for our second experiment. The response variable (respondents’ willingness to follow protected area rules) was condensed to a 3-point scale, and the 2 vignette factors (corruption and descriptive norms) were included as predictors alongside respondent age, gender, years of formal education completed, household multidimensional poverty index (MPI), and attitude toward protected area rules. Following Christensen (2019), our nonhierarchical models were checked to assess nonproportional odds assumptions, with scale effects included where appropriate. We selected scale effects over nominal effects because these offer greater flexibility for all values of predictor variables and use fewer parameters, leading to more sensitive tests than those that include nominal effects (Christensen, 2019). The data that support the findings of this study are openly available in Figshare at http://doi.org/10.6084/m9.figshare.25772064.

## Results

### Respondent demographics

Samples in both countries were almost evenly divided by gender (Indonesia, 52% male; Tanzania, 51%). The median years of completed formal education were higher in Indonesia (9, interquartile range [IQR] 6−12) than Tanzania (7, IQR 7−7), and mean age of respondents was 36 years (minimum 18, maximum 60) in Indonesia and 37 in Tanzania (minimum 18, maximum 70). Overall, households were poorer in Tanzania (MPI [0, low levels of poverty; 1, poverty across all dimensions measured] median 0.22, IQR 0.11−0.27) than Indonesia (median 0.05, IQR 0−0.05). In Indonesia, 71% of respondents agreed or strongly agreed that the rules of Gunung Leuser National Park were fair and consistent with the law. In Tanzania, 96% of respondents similarly agreed or strongly agreed.

### Factors affecting perceived fairness of sanctions (Experiment 1)

**Figure 2:**
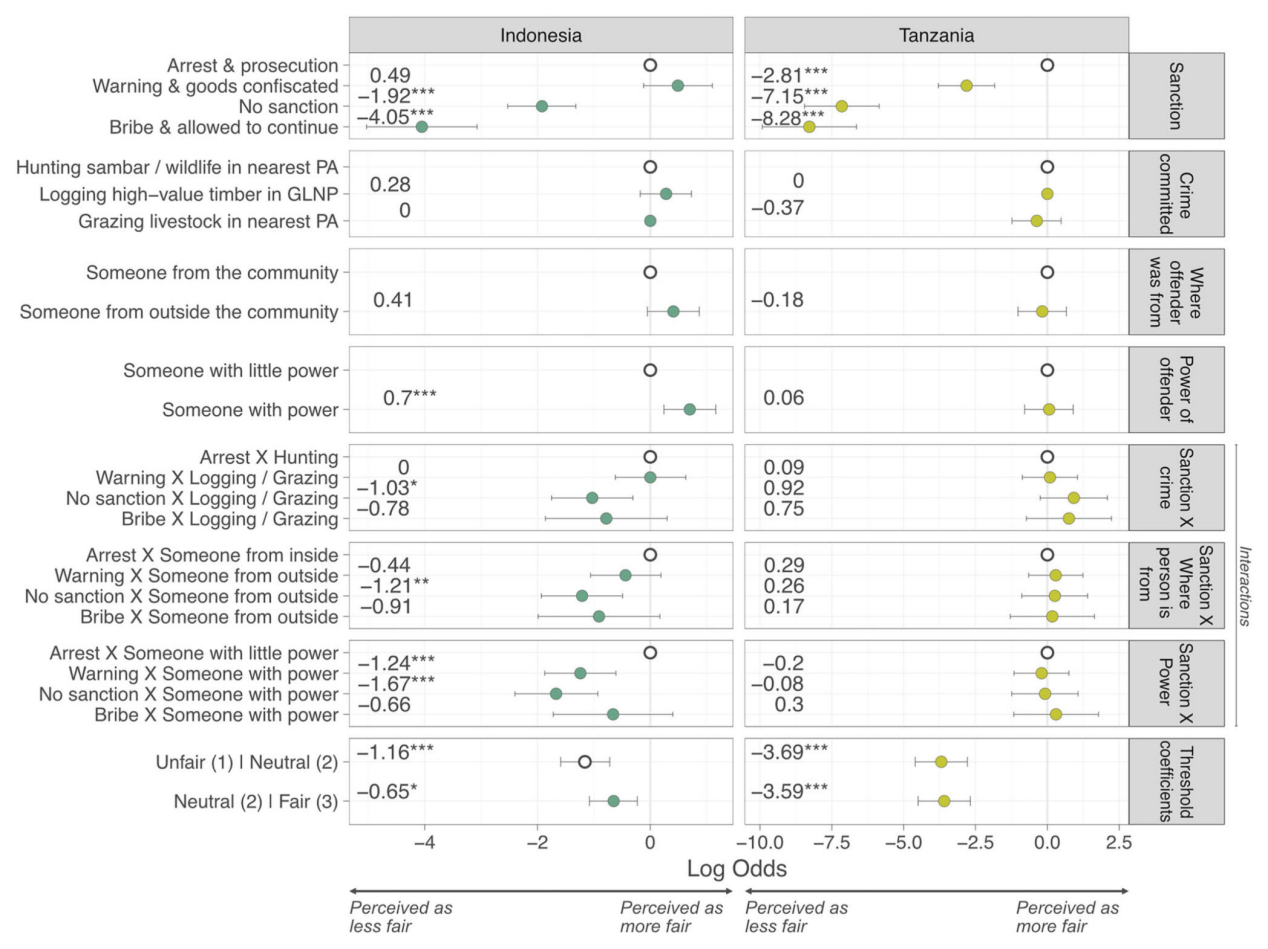
Multilevel ordinal regression modeling of the perceived fairness of different sanctions for violations of protected area rules, where response is ona 3-point Likert scale (1, unfair, to 3, fair) (GLNP, Gunung Leuser National Park; PA, protected area; white circles, reference categories; ***p < 0.001, **p < 0.01, *p < 0.05; error bars, 95% confidence intervals). Table of results is in Appendix S2.

At both sites, the most significant factor affecting respondents’ perceptions of fairness was the sanction administered (Figure 2). In Indonesia, issuing a warning and confiscating goods was perceived as fairer than arrest and prosecution, but not significantly so (*β* = 0.49, *p* = 0.116). Conversely, failing to administer a sanction (*β* = −1.92, *p* ≤ 0.001) and a bribe being accepted with the transgression allowed to continue (*β* = −4.05, *p* ≤ 0.001) were both perceived as significantly less fair than arrest and prosecution. The same pattern applied in Tanzania; however, the distribution of a warning with goods being confiscated (*β* = −2.81, *p* ≤ 0.001) was seen as significantly less fair than arrest and prosecution. Interestingly, in Indonesia, scenarios involving someone with lots of power were perceived as significantly fairer than those involving someone with little power (*β* = 0.70, *p* = 0.003), perhaps illustrating respondents perceived it fairer when enforcers apprehend powerful people compared to less powerful. When interactions were included, it was perceived as less fair if a powerful person received a warning (*β* = −1.24, *p* ≤ 0.001) or no sanction (*β* = −1.67, *p* ≤ 0.001) rather than being arrested and prosecuted. A significant negative interaction was identified if someone from outside the community went unsanctioned (*β* = 1.21, *p* = 0.007), suggesting respondents thought it particularly unfair if people from outside the community were treated more leniently. A significant negative interaction was also identified if no sanction was administered when an offender was caught logging (*β* = 1.03, *p* = 0.05), suggesting respondents felt that inaction by law enforcers was less appropriate for logging, compared to hunting. No significant interactions were identified in Tanzania.

### How corruption and descriptive norms affect willingness to comply with protected area rules (Experiment 2)

**Figure 3:**
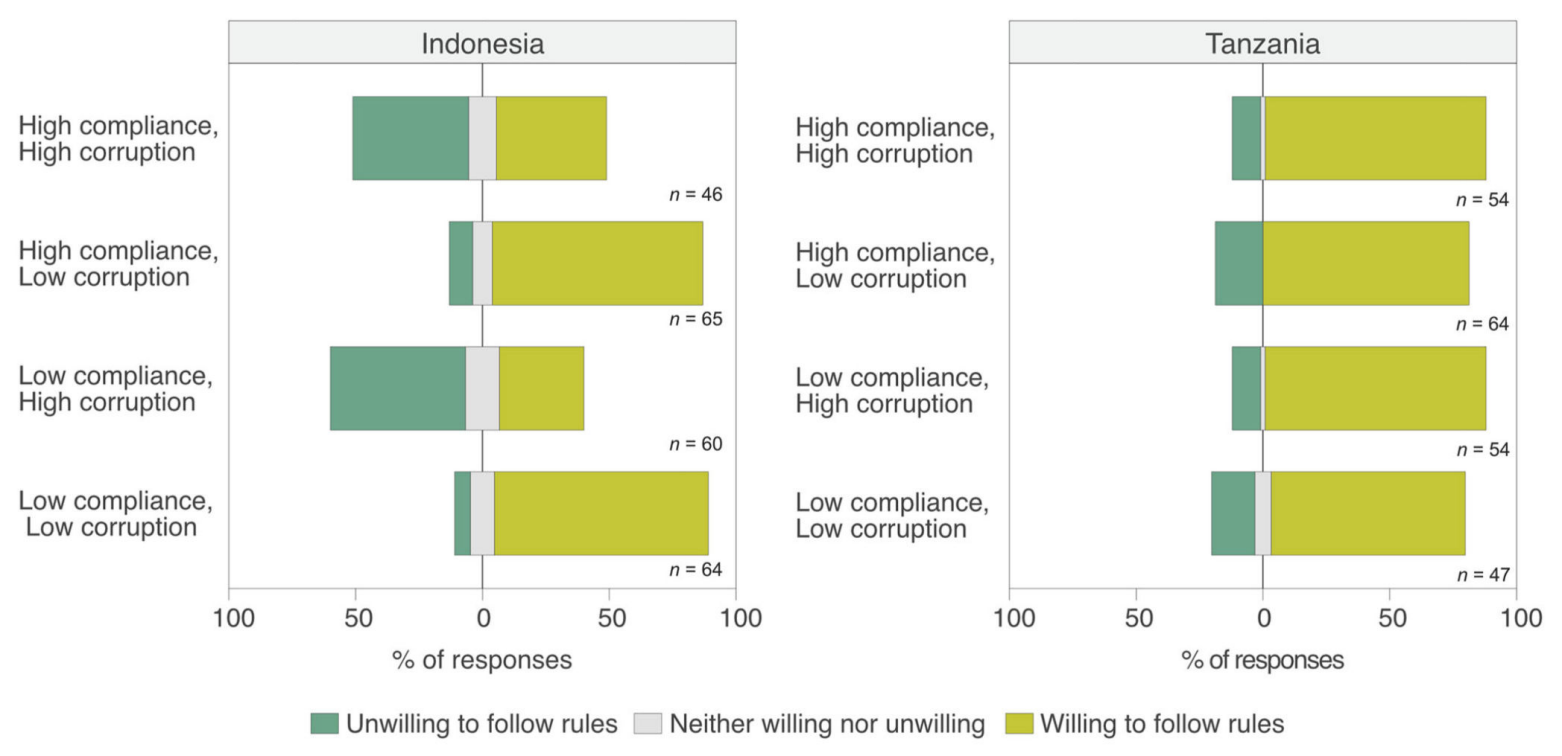
Distribution of responses to the query how willing are you to follow protected area rules under different scenarios.

Overall, respondents in Tanzania reported higher willingness to follow rules than in Indonesia (Figure 3). In both countries, modeling suggested that those with more positive attitudes toward protected area rules were more willing to comply (Indonesia: *β* = 0.76, *p* = 0.014; Tanzania: *β* = 0.38, *p* = 0.029) (Table 3). The impact of descriptive norms and corruption on willingness to follow rules differed across countries. In Indonesia, descriptive norms did not significantly affect individuals’ willingness to follow protected area rules (*β* = −0.07, *p* = 0.875), whereas corruption did (*β* = −2.49, *p* ≤ 0.001); respondents were less willing to follow rules if a bribe was likely to be accepted. In Tanzania, corruption did not affect individuals’ willingness to follow protected area rules (*β* = 0.27, *p* = 0.199), but descriptive norms did (*β* = −0.97, *p* = 0.007). In scenarios where community compliance was high, willingness to follow rules was also high. In Tanzania, gender (*β* = −1.03, *p* = 0.012) and age (*β* = 0.03, *p* = 0.03) were significant predictors, with female and older respondents more willing to comply. Years of formal education completed and the household’s multidimensional poverty status did not affect willingness to follow rules in either country.

## Discussion

Our results demonstrate that how authorities administered sanctions around protected areas mattered. In Tanzania, regardless of where an offender was from or the power the person held, respondents expressed a clear preference that authorities administer sanctions consistently and in line with the law. Similarly, in Indonesia, respondents expected powerful actors to be treated the same as those without power—a sentiment supported elsewhere (Gore et al., 2013). This is important because powerful actors, such as those with significant social or financial standing, can sometimes use their advantage to influence criminal justice processes, resulting in outcomes where disadvantaged groups incur more severe punishments, whereas well-funded or well-connected offenders do not (Wilson & Boratto, 2020). Such administration can alienate local people, generate hostility, and lead to general distrust of protected area authorities and may encourage further rule breaking or conflict (Moreto & Gau, 2017; von Essen et al., 2014). To this end, there have been calls for conservationists to pay greater attention to the role of procedural justice within conservation, both more broadly (Ruano-Chamorro et al., 2022) and specifically within conservation law enforcement (F.S.J., personal observation). It is theorized that fair treatment of individuals by authorities, for example, through consistent, even-handed enforcement of laws, can enhance people’s belief in the legitimacy of authorities, which in turn can foster greater voluntary compliance, reduce law enforcement costs, and improve relations between protected area authorities and local people (Moreto & Gau, 2017; Stern, 2008; Tyler, 2021).

The way in which discretion is exercised is also important (Eliason, 2021). In Indonesia, it was seen as significantly less fair if an outsider received more lenient sanctions than a community member. Failure to differentiate between outsiders’ rule breaking for criminal purposes and local people doing so for subsistence needs can generate resentment of authorities among local people (Bell et al., 2007). Our findings from Indonesia suggest a social expectation for law enforcers to exercise discretion compassionately (Belecky, Moreto, et al., 2021; Paley, 2015). Such expectations may be greater and more likely to be upheld in contexts where law enforcers police their own communities, particularly if failure to exercise discretion could result in social repercussions for the enforcers or their families (e.g., Bell et al., 2007; Dutta, 2020). These social expectations were not detected in Tanzania, potentially reflecting variation in the rules governing natural resource use in the 2 study landscapes, as well as their implementation. In Tanzania, hunting any wildlife or entering a national park or game reserve without a permit is prohibited. Most people surveyed reported that the rules were fair and consistent with the law, complimenting other research that shows people living here know the rules and believe they would be enforced by authorities (Ibbett et al., 2023). Elsewhere, studies show top-down models of enforcement, similar to those in Tanzania, leave limited scope for law enforcers to use discretion (Day et al., 2023), which may go some way to explaining this finding. In contrast, in the Leuser ecosystem, there is a complex array of protected area designations, including areas that allow resource extraction. Local knowledge of rules is generally lower (Ibbett et al., 2023), with fewer people agreeing that rules were fair and consistent with the law. Although widely studied in policing, discretion remains underexplored in conservation, particularly in the Global South (although see Warchol & Kapla [2012]). However, much could be learned by studying how discretion influences the legitimacy with which protected area authorities are viewed.

Despite the fact paying bribes dilutes the costs of being caught (bribes usually cost less than fines or incarceration) (Polinsky & Shavell, 2001), bribery was never considered fair, and in Indonesia, such corruption significantly undermined people’s willingness to follow rules. Corruption undermines trust in authorities (Jackson et al., 2014), making governance harder and less effective (Kahler & Gore, 2012). Resolving corruption in conservation law enforcement is challenging; it is a multifaceted and complex problem that manifests in many ways (Robbins, 2000). Ensuring the basic needs and welfare of individual law enforcers are met, for example, through prompt and sufficient pay, and adequate provisioning, may help reduce incentives for corruption (Belecky, Parry Jones, et al., 2021), although fair administration of the law, particularly when dealing with powerful offenders, can also be difficult to enact. In some circumstances, physical, psychological, or financial threats may make it safer for law enforcers to collude (Miller, 2011), and in contexts where corruption is systemic and institutionalized or where positions are maintained through patronage, pressure from superiors may make it impossible to avoid (Belecky, Moreto, et al., 2021; Paley, 2015). The development of well-targeted anticorruption policies thus requires an understanding of the determinants of and relationships that exist between different actors, alongside simultaneous reform of political systems and enforcement infrastructure (Wilson & Damania, 2005). Despite the need to better understand how corruption hampers law enforcement and conservation more broadly, it remains poorly studied within conservation science (Sundström, 2016), and there is considerable scope for further investigation.

In Tanzania, descriptive norms were a stronger predictor of people’s willingness to comply with protected area rules than corruption. This supports previous research reporting significant social disapproval of rule breaking and bribery at the study site (Ibbett et al., 2023) and that fear of informal sanctions (e.g., social ostracization) can be more costly than formal punishment (Atuo et al., 2020). Elsewhere, social norms and their repercussions have been reported to both regulate people’s adherence to rules and the sharing of information regarding crimes. For example, in Uganda, Anagnostou et al. (2020) found that those who repeatedly ignore neighbors’ requests to stop rule breaking are more likely to be exposed during meetings with community outreach rangers. The finding that age and gender were significant predictors of willingness to comply in Tanzania was unsurprising; others have also found these characteristics to be associated with rule breakers in the landscape (Hariohay et al., 2019). In neither landscape did the multidimensional poverty status of respondents’ household predict their propensity to comply with protected area rules, highlighting that the drivers of noncompliance are often more complex than poverty alone (Duffy et al., 2015).

In both countries, we found positive associations between respondents’ attitudes toward protected area rules and their willingness to comply, suggesting that fair enforcement of rules is only part of the picture. Higher compliance is also likely affected by whether the rules themselves are perceived to be just and fair (Ruano-Chamorro et al., 2022). For example, in South Africa, the concept of contested illegality, where legitimacy of the rules themselves is questioned, affects the actions of those convicted of wildlife offences (Hübschle, 2017a). This concept is important because in many protected areas worldwide, the rules of governance in place today remain largely unchanged from those imposed by colonial administrations, which specifically aimed to separate local people from nature and to control resources, people, and territories (Bontempi et al., 2023; Igoe & Brockington, 2007).

Although our factorial survey experiment offers valuable insights into peoples’ perceptions of law enforcement administration across 2 different contexts, it was not without limitations. First, our design was relatively simplistic. We explored few variables, yet many other factors influence people’s perspectives and subsequent compliance (Bell et al., 2007; Robbins et al., 2005; von Essen, 2014). Because of their hypothetical nature, vignettes may have been difficult for participants to understand, and the illicit and thus sensitive nature of the topic may have made some respondents wary of providing honest answers (Ibbett et al., 2023). Further, in the second experiment, specifying “…law enforcers will accept a ‘small’ amount of money…” may have been unnecessary. Stating law enforcers will accept money would have been sufficient to illustrate the act of bribery. Moreover, we did not attempt to formally measure people’s perceptions of procedural justice or authorities legitimacy based on frameworks outlined in criminal psychology (Tyler, 2021); doing so would strengthen the ability of conservation scientists to suggest practical measures, including tailored training programs (e.g., Gilbert et al., 2015). Additionally, our research only examined the perspectives of those who might experience law enforcement. Research into rangers’ perspectives is still an emerging area of conservation scholarship (Moreto et al., 2021), which would benefit from increased attention.

Significant conservation resource is invested in enforcing compliance with protected area rules, with increasing calls emphasizing the need for more law enforcement personnel to meet global protected area targets (Appleton et al., 2022). Yet, our experiments, conducted across 2 contexts, suggest that more boots on the ground alone will be insufficient to generate greater compliance. Conservation policy and practice must also address challenges associated with fair administration of protected area laws. The need to professionalize conservation law enforcement, to tackle issues such as corruption, and to promote healthy relationships between law enforcers and local people is widely recognized (Appleton et al., 2021; Dutta, 2020; Woodside et al., 2021), yet remains poorly addressed. Although the ratification and adoption of professional codes of conduct for rangers (International Ranger Federation, 2021), the increased emphasis on the need for human-rights-based training for law enforcers (Palama & Martin, 2023), and the introduction of social safeguards and grievance mechanisms represent positive steps, there is still much more work to do. Indeed, in contexts where protected area establishment is associated with structural injustices of colonialism, including marginalization and dispossession, professionalization of the ranger force alone is unlikely to increase compliance or the perceived legitimacy of enforcers and their rules. Moving forward, conservation policy and practice may have much to learn from pilot projects that are incorporating the principles of environmental restorative justice as a way of moving beyond punitive enforcement measures (Hübschle et al., 2021). Such efforts involve stakeholders actively engaging in processes that acknowledge the harms and wrongdoing inflicted by structural, systemic, and individual injustices (Forsyth et al., 2021). When combined, these efforts could provide a stronger foundation on which to administer laws and improve compliance, securing fairer outcomes for people and nature living in and around protected areas.

## Supplementary Material

Appendix

## Figures and Tables

**Table 1:** Factors and their levels that formed the vignettes applied in factorial survey Experiment 1 to assess how fair respondents living around protected areas in Indonesia and Tanzania perceived sanctioning by law enforcers to be.

Factor	Level
Crime committed	Indonesia only
	Hunting protected sambar in the national park;
	Logging high-value timber in the national park
	Tanzania only
	Hunting wildlife in the nearest protected area;
	Grazing livestock in the nearest protected area
Where offenderwas from	Someone from the community
	Someone from outside the community
Power held by offender	Someone with little power
	Someone with power
Sanction administered	Arrest and prosecution (a lawful sanction)
	Warning with resources confiscated (a lawful sanction)
	No repercussions for offender (representing use of discretion by the law enforcer)
	Bribe accepted by law enforcer and offender allowed to continue (representing an abuse of power by the law enforcer)

**Table 2:** For the second experiment, one of 4 vignettes^a^ was randomly allocated among respondents, and they were asked how willing they would be to follow protected area rules if the given scenario were true.

Descriptive norms (compliance of others)	Corruption (likelihood of a bribe being accepted)	
	High	Low
High	Your fellow community members rarely^b^ break rules and enter the protected area to collect resources. If caught breaking rules, it is highly likely^b^ that law enforcers will accept a small amount of money, and any criminal charges or fines will disappear.	Your fellow community members rarely^b^ break rules and enter the Protected Area to collect resources. If caught breaking rules, it is highly unlikely^b^ that law enforcers will accept a small amount of money, resulting in criminal charges or fines.
Low	Your fellow community members often^b^ break rules and enter the Protected Area to collect resources. If caught breaking rules, it is highly likely^b^ that law enforcers will accept a small amount of money, and any criminal charges or fines will disappear.	Your fellow community members often^b^ break rules and enter the Protected Area to collect resources. If caught breaking rules, it is highly unlikely^b^ that law enforcers will accept a small amount of money, resulting in criminal charges or fines.

a Vignettes were composed of 2 factors, each with 2 levels.

b Differences in vignettes.

**Table 3:** Ordinal regression modeling of respondents’ willingness to follow protected area rules under different scenarios (3, willing to follow rules; 2, neither willing nor unwilling to follow rules; 1, unwilling to follow rules).

Predictor		Indonesia			Tanzania		
		Log odds	95% CI	*p*		Log odds	95% CI	*p*
Descriptive norms (compliance of others)	High^a^	–	–	–		–	–	–
	Low	−0.07	−0.93 to 0.79	0.875		−0.97	−1.67 to −0.27	0.007^b^
Corruption (acceptance of bribe)	Low^a^	–	–	–		–	–	–
	High	−2.49	−3.55 to −1.43	<0.001^b^		0.27	−0.14 to 0.69	0.199
Gender	Female^a^	–	–	–		–	–	–
	Male	0.23	−0.67 to 1.13	0.612		−1.03	−1.84 to −0.23	0.012^b^
Age		0.05	−0.01 to 0.10	0.083		0.03	0.00−0.05	0.030
Years of formal education completed		0.04	−0.11 to 0.18	0.620		0.03	−0.05 to 0.10	0.473
Household multidimensional poverty^c^		−0.37	−6.75 to 6.00	0.909		−1.74	−3.86 to 0.38	0.108
Attitude toward protected area rules^d^		0.76	0.16−1.36	0.014^b^		0.38	0.04−0.72	0.029^b^
Scale coefficients								
Level of compliance	Low	–	–	–		−1.12	−1.67 to −0.27	0.010^b^
Level of corruption	High	0.75	−3.55 to −1.43	0.040		–	–	–
Threshold coefficients								
Unwilling (1) | Neutral (2)		2.13	−1.52 to 5.79	0.252		0.12	−1.70 to 1.94	0.897
Neutral (2) | Willing (3)		3.15	−0.68 to 6.99	0.107		0.24	−1.61 to 2.09	0.800
Observations		221				211		
Log likelihood		−163.17				−89.97		
Akaike information criterion		346.35				199.94		

a Reference category.

b Variable identified as significant.

c Multidimensional poverty index, range 0−1 (0, low levels of poverty; 1, poverty across all measured dimensions).

d Measured as agreement with the following statement: “The rules of the protected area are fair and consistent with the law.”
