# Supplementary material for: Improving compliance around protected areas through fair administration of rules
Source: Conserv Biol. 2024 Jul 17;39(1):e14332. doi: 10.1111/cobi.14332 (PMC7617185; doi:10.1111/cobi.14332)
Supplement: Supplementary file 1 — Supplementary Materials [file COBI-39-e14332-s001.docx]

Supplementary Materials

Table of Contents

[Appendix 1. Experimental design & survey instruments 1](#_Toc127542230)

[Calculation of Multidimensional Poverty Indices (MPI) 1](#_Toc127542231)

[Factorial Survey Experiment (FSE) Design 2](#_Toc127542232)

[Vignette Descriptions 4](#_Toc127542233)

[Indonesian Survey Instrument 8](#_Toc127542234)

[Tanzanian Survey Instrument 15](#_Toc127542235)

[Appendix 2. Analyses 21](#_Toc127542236)

[Ordinal Regression Analyses 21](#_Toc127542237)

[Analysis of data from Indonesia for Experiment 1 *(Fairness of sanctions)* 21](#_Toc127542238)

[Analysis of data from Tanzania for Experiment 1 *(Fairness of sanctions)* 23](#_Toc127542239)

[Analysis of data from Indonesia for Experiment 2 24](#_Toc127542240)

[Analysis of data Tanzania for Experiment 2 26](#_Toc127542241)

## Appendix 1. Experimental design & survey instruments

### Calculation of Multidimensional Poverty Indices (MPI)

Table A1.1. Table outlining how a Multidimensional Poverty Index was calculated for each respondent’s household. The classifications follow Table 1 of Global MPI Methodological Note 51 (Alkire et al. 2021), and where appropriate were adapted for the local context. Responses to each question were coded 1 (signalling deprivation) or 0 (signalling no deprivation), and then multiplied by the weightings shown. Multidimensional Poverty Indices were calculated by summing all weightings across all indicators for each respondent.

| **Poverty Indicator** | **Measure** | **Weight allocated to measure** | **A household was considered deprived (and allocated a score of 1) where… (Based on Alkire et al. 2016; 2021)** | **Notes** |
| --- | --- | --- | --- | --- |
| Education (1/3) | Years of Schooling | 1/6  (0.1667) | No eligible household member has completed six years of schooling |  |
|  | Child school attendance | 1/6  (0.1667) | Any school-aged child is not attending school up to the age at which he/she would complete class 8. |  |
| Health  (1/3) | Nutrition HHS | 1/6  (0.1667) | Any person under 70 years of age for whom there is nutritional information is undernourished.  Measured using Household Hunger Score from FANTA USAID toolkit, which asks three questions:  • In the past 12 months, how often was there no food to eat of any kind in your house because of lack of resources to get food?  • In the past 12 months, how often did any household member go to sleep at night hungry because there was not enough food?  • In the past 12 months, how often did any household member go a whole day and night without eating anything because there was not enough food? | *In Indonesia:*  A household measured as deprived if their answer to any of the three questions was one day.  *In Tanzania:*  A household measured as deprived if their answer to any of the three questions was +30days. |
|  | Child Mortality | 1/6  (0.1667) | A child under 18 has died in the household in the five-year period preceding the survey |  |
| Living standards (1/3) | Cooking Fuel | 1/18  (0.055) | A household cooks with dung, agricultural crop, shrubs, wood, charcoal or coal. |  |
|  | Sanitation | 1/18  (0.055) | The household has unimproved or no sanitation facility or it is improved but shared with other households. | A household is considered to have access to improved sanitation if it has some type of flush toilet or latrine, or ventilated improved pit or composting toilet, if they are not shared. |
|  | Drinking water | 1/18  (0.055) | The household does not have access to improved drinking water (according to SDG guidelines) or safe drinking water is at least a 30-minute walk from home, roundtrip. | A household has access to clean drinking water if the water source is any of the following types: piped water, public tap, borehole or pump, protected well, protected spring, or rainwater, and it is within a 30-minute round trip by usual means of transport.  Bottled water included as an improved water source here following notes in Alkire 2021 |
|  | Electricity | 1/18  (0.055) | The household has no electricity. |  |
|  | Housing | 1/18  (0.055) | The household has a dirt, sand, dung or ‘other’ (unspecified) type of floor |  |
|  | Assets | 1/18  (0.055) | The household does not own more than one radio, TV, telephone, bicycle, motorbike or refrigerator and does not own a car or truck. | Deprived: Sum of the number of small assets is less than two AND no car/truck  Not deprived: Sum of small assets greater than 1 OR owns a car/truck |

*Alkire, S., Jindra, C., Robles, G. and Vaz, A. (2016). “Multidimensional Poverty Index - 2016: Brief methodological note and results.” OPHI Briefing 42, University of Oxford.* [*https://www.ophi.org.uk/wp-content/uploads/OPHIBrief_42_MPI_meth_note_2016.pdf*](https://www.ophi.org.uk/wp-content/uploads/OPHIBrief_42_MPI_meth_note_2016.pdf)

*Alkire, S., Kanagaratnam, U., and Suppa, N. (2021). ‘The global Multidimensional Poverty Index (MPI) 2021’, OPHI MPI Methodological Note 51, Oxford Poverty and Human Development Initiative, University of Oxford.* [*https://www.ophi.org.uk/wp-content/uploads/OPHI_MPI_MN_51_2021_4_2022.pdf*](https://www.ophi.org.uk/wp-content/uploads/OPHI_MPI_MN_51_2021_4_2022.pdf)

### Factorial Survey Experiment (FSE) Design

To calculate the total number of vignette combinations (i.e., the full factorial design) we multiplied the number of levels in each factor (e.g., 2 x 2 x 2 x 4 = 32). Ideally, respondents would be asked to assess all 32 vignettes. However, presenting a respondent with too many vignettes’ risks fatigue, boredom and unwanted methodological effects. Instead, a common approach is to divide the vignettes into smaller ‘blocks’ or sets of vignettes, a selection of which are administered to respondents (Ausperg & Hinz 2015). Vignette allocation to blocks can be random or systematic. Most studies use random allocation, however, increasingly it is seen as good practice (and more statistically efficient) to use systematic designs (Dülmer 2016). Specialist software can be used to develop the most efficient combination of blocks. Here, using the software SAS and following the code of Lawson et al. (2009) (below) we used a mixed-level full factorial design, separated into four blocks of eight vignettes (Table A1.2). This design has a D-efficiency of 100, and is orthogonal (meaning all main effects and interaction effects can be estimated independently of all other effects) and balanced (each level occurs equally often within each vignette) (Dülmer 2016). Respondents were randomly allocated to a block, and the vignette order within each block was randomised.

Table A1.2. Blocked full-factorial design for 2x2x2x4 with a D-efficiency of 100. The top row shows the four factors (crime committed, power of offender, where offender was from, and sanction administered) and the columns show the levels of this factor allocated in each vignette. In Tanzania, the crime ‘logging’ was replaced with ‘grazing livestock’.

| **Block ID** | **Vignette ID** | **Crime committed** | **Power of offender** | **Where offender was from** | **Sanction administered** |
| --- | --- | --- | --- | --- | --- |
| 1 | 1 | logging | big | outsider | nothing |
| 1 | 2 | hunting | small | outsider | bribe |
| 1 | 3 | hunting | big | outsider | warning |
| 1 | 4 | logging | small | insider | warning |
| 1 | 5 | hunting | big | insider | arrest |
| 1 | 6 | logging | small | outsider | arrest |
| 1 | 7 | hunting | small | insider | nothing |
| 1 | 8 | logging | big | insider | bribe |
| 2 | 9 | hunting | small | outsider | nothing |
| 2 | 10 | logging | big | outsider | arrest |
| 2 | 11 | logging | big | insider | nothing |
| 2 | 12 | logging | small | outsider | warning |
| 2 | 13 | hunting | big | outsider | bribe |
| 2 | 14 | hunting | big | insider | warning |
| 2 | 15 | hunting | small | insider | arrest |
| 2 | 16 | logging | small | insider | bribe |
| 3 | 17 | hunting | small | outsider | warning |
| 3 | 18 | hunting | big | outsider | arrest |
| 3 | 19 | logging | big | insider | warning |
| 3 | 20 | logging | small | insider | arrest |
| 3 | 21 | hunting | big | insider | nothing |
| 3 | 22 | hunting | small | insider | bribe |
| 3 | 23 | logging | small | outsider | nothing |
| 3 | 24 | logging | big | outsider | bribe |
| 4 | 25 | logging | big | insider | arrest |
| 4 | 26 | logging | small | outsider | bribe |
| 4 | 27 | hunting | big | insider | bribe |
| 4 | 28 | logging | big | outsider | warning |
| 4 | 29 | hunting | big | outsider | nothing |
| 4 | 30 | logging | small | insider | nothing |
| 4 | 31 | hunting | small | outsider | arrest |
| 4 | 32 | hunting | small | insider | warning |

[https://welcome.oda.sas.com](https://welcome.oda.sas.com/login)

SAS Code:

proc plan;

factors actor=2 power=2 behaviour=2 sanction=4;

output out=cdesign actor cvals=('insider' 'outsider')

power cvals=('small' 'big')

behaviour cvals=('hunting' 'logging')

sanction cvals=('nothing' 'warning' 'bribe' 'arrest');

proc optex data=cdesign coding=orthcan seed=73565;

class actor power behaviour sanction;

model actor power behaviour sanction actor*behaviour actor*sanction actor*power behaviour*power sanction*power behaviour*sanction;

blocks structure=(4)8 init=chain noexchange;

generate initdesign=cdesign method=sequential;

output out=bdesign blockname=blk; run;

proc print data=bdesign; run;

### Vignette Descriptions

Table A1.3. Full vignette descriptions and their Bahasa Indonesia translations used in Experiment 1 – which assessed the perceived fairness of sanctions

| **Block** | **Vignette** | **English** | **Bahasa Indonesia** |
| --- | --- | --- | --- |
| 1 | 1 | 1. A powerful person from outside your community is caught logging in TNGL by a ranger. The ranger allowed them to continue without any repercussions. | 1. Seseorang yang memiliki kekuasaan dari luar komunitas Anda ketahuan menebang pohon di TNGL oleh ranger. Ranger memperbolehkan mereka untuk melanjutkan tanpa konsekuensi. |
| 1 | 2 | 2. A person with no power from outside your community is caught hunting a protected sambar in TNGL by a ranger. The ranger accepted a bribe and they were allowed to continue. | 2. Seseorang yang tidak memiliki kekuasaan dari luar komunitas Anda ketahuan berburu rusa yang dilindungi di TNGL oleh ranger. Ranger menerima sogokan dan mereka diperbolehkan untuk melanjutkan. |
| 1 | 3 | 3. A powerful person from outside your community is caught hunting a protected sambar in TNGL by a ranger. The ranger confiscated the sambar, and warned them not to hunt again. | 3. Seseorang yang memiliki kekuasaan dari luar komunitas Anda ketahuan berburu rusa yang dilindungi di TNGL oleh ranger. Ranger menyita barang bukti (contohnya: rusa dan alat berburu) dan memperingatkan mereka untuk tidak berburu lagi. |
| 1 | 4 | 4. A person with no power from your community is caught logging in TNGL by a ranger. The ranger confiscated the timber and warned them not to log again. | 4. Seseorang yang tidak memiliki kekuasaan dari komunitas Anda ketahuan menebang pohon di TNGL oleh ranger. Ranger menyita barang bukti (contohnya: kayu dan gergaji mesin) dan memperingatkan mereka untuk tidak menebang lagi. |
| 1 | 5 | 5. A powerful person from your community is caught hunting a protected sambar in TNGL by a ranger. The ranger arrested them and they were prosecuted. | 5. Seseorang yang memiliki kekuasaan dari komunitas Anda ketahuan berburu rusa yang dilindungi di TNGL oleh ranger. Ranger menangkap mereka dan mereka diproses hukum. |
| 1 | 6 | 6. A person with no power from outside your community is caught logging in TNGL by a ranger. The ranger arrested them and they were prosecuted. | 6. Seseorang yang tidak memiliki kekuasaan dari luar komunitas Anda ketahuan menebang pohon di TNGL oleh ranger. Ranger menangkap mereka dan mereka diproses hukum. |
| 1 | 7 | 7. A person with no power from your community is caught hunting a protected sambar in TNGL by a ranger. The ranger allowed them to continue without any repercussions. | 7. Seseorang yang tidak memiliki kekuasaaan dari komunitas Anda ketahuan berburu rusa yang dilindungi di TNGL oleh ranger. Ranger memperbolehkan mereka untuk melanjutkan tanpa ada tindak lanjut. |
| 1 | 8 | 8. A powerful person from your community is caught logging in TNGL by a ranger. The ranger accepted a bribe and they were allowed to continue. | 8. Seseorang yang memiliki kekuasaan dari komunitas Anda ketahuan menebang pohon di TNGL oleh ranger. Ranger menerima sogokan mereka dan mereka diperbolehkan untuk melanjutkan. |
| 2 | 9 | 9. A person with no power from outside your community is caught hunting a protected sambar in TNGL by a ranger. The ranger allowed them to continue without any repercussions. | 9. Seseorang yang tidak memiliki kekuasaaan dari luar komunitas Anda ketahuan berburu rusa yang dilindungi di TNGL oleh ranger. Ranger memperbolehkan mereka untuk melanjutkan tanpa konsekuensi. |
| 2 | 10 | 10. A powerful person from outside your community is caught logging in TNGL by a ranger. The ranger arrested them and they were prosecuted. | 10. Seseorang yang memiliki kekuasaan dari luar komunitas Anda ketahuan menebang pohon di TNGL oleh ranger. Ranger menangkap mereka dan mereka diproses hukum. |
| 2 | 11 | 11. A powerful person from your community is caught logging in TNGL by a ranger. The ranger allowed them to continue without any repercussions. | 11. Seseorang yang memiliki kekuasaan dari komunitas Anda ketahuan menebang pohon di TNGL oleh ranger. Ranger memperbolehkan mereka untuk melanjutkan tanpa konsekuensi. |
| 2 | 12 | 12. A person with no power from outside your community is caught logging in TNGL by a ranger. The ranger confiscated the timber and warned them not to log again. | 12. Seseorang yang tidak memiliki kekuasaan dari luar komunitas Anda ketahuan menebang pohon di TNGL oleh ranger. Ranger menyita barang bukti (contohnya: kayu dan gergaji mesin) dan memperingatkan mereka untuk tidak menebang lagi. |
| 2 | 13 | 13. A powerful person from outside your community is caught hunting a protected sambar in TNGL by a ranger. The ranger accepted a bribe and they were allowed to continue. | 13. Seseorang yang memiliki kekuasaan dari luar komunitas Anda ketahuan berburu rusa yang dilindungi di TNGL oleh ranger. Ranger menerima sogokan dan mereka diperbolehkan untuk melanjutkan. |
| 2 | 14 | 14. A powerful person from your community is caught hunting a protected sambar in TNGL by a ranger. The ranger confiscated the sambar, and warned them not to hunt again. | 14. Seseorang yang memiliki kekuasaan dari komunitas Anda ketahuan berburu rusa yang dilindungi di TNGL oleh ranger. Ranger menyita barang bukti (contohnya: rusa dan alat berburu) dan memperingatkan mereka untuk tidak berburu lagi. |
| 2 | 15 | 15. A person with no power from your community is caught hunting a protected sambar in TNGL by a ranger. The ranger arrested them and they were prosecuted. | 15. Seseorang yang tidak memiliki kekuasaan dari komunitas Anda ketahuan berburu rusa yang dilindungi di TNGL oleh ranger. Ranger menangkap mereka dan mereka diproses hukum. |
| 2 | 16 | 16. A person with no power from your community is caught logging in TNGL by a ranger. The ranger accepted a bribe and they were allowed to continue. | 16. Seseorang yang tidak memiliki kekuasaan dari komunitas Anda ketahuan menebang pohon di TNGL oleh ranger. Ranger menerima sogokan dan mereka diperbolehkan untuk melanjutkan. |
| 3 | 17 | 17. A person with no power from outside your community is caught hunting a protected sambar in TNGL by a ranger. The ranger confiscated the sambar, and warned them not to hunt again. | 17. Seseorang yang tidak memiliki kekuasaan dari luar komunitas Anda ketahuan berburu rusa yang dilindungi di TNGL oleh ranger. Ranger menyita barang bukti (contohnya: rusa dan alat berburu) dan memperingatkan mereka untuk tidak berburu lagi. |
| 3 | 18 | 18. A powerful person from outside your community is caught hunting a protected sambar in TNGL by a ranger. The ranger arrested them and they were prosecuted. | 18. Seseorang yang memiliki kekuasaan dari luar komunitas Anda ketahuan berburu rusa yang dilindungi di TNGL oleh ranger. Ranger menangkap mereka dan mereka diproses hukum. |
| 3 | 19 | 19. A powerful person from your community is caught logging in TNGL by a ranger. The ranger confiscated the timber and warned them not to log again. | 19. Seseorang yang memiliki kekuasaan dari komunitas Anda ketahuan menebang pohon di TNGL oleh ranger. Ranger menyita barang bukti (contohnya: kayu dan gergaji mesin) dan memperingatkan mereka untuk tidak menebang lagi. |
| 3 | 20 | 20. A person with no power from your community is caught logging in TNGL by a ranger. The ranger arrested them and they were prosecuted. | 20. Seseorang yang tidak memiliki kekuasaan dari komunitas Anda ketahuan menebang pohon di TNGL oleh ranger. Ranger menangkap mereka dan mereka diproses hukum. |
| 3 | 21 | 21. A powerful person from your community is caught hunting a protected sambar in TNGL by a ranger. The ranger allowed them to continue without any repercussions. | 21. Seseorang yang memiliki kekuasaaan dari komunitas Anda ketahuan berburu rusa yang dilindungi di TNGL oleh ranger. Ranger memperbolehkan mereka untuk melanjutkan tanpa konsekuensi. |
| 3 | 22 | 22. A person with no power from your community is caught hunting a protected sambar in TNGL by a ranger. The ranger accepted a bribe and they were allowed to continue. | 22. Seseorang yang tidak memiliki kekuasaan dari komunitas Anda ketahuan berburu rusa yang dilindungi di TNGL oleh ranger. Ranger menerima sogokan dan mereka diperbolehkan untuk melanjutkan. |
| 3 | 23 | 23. A person with no power from outside your community is caught logging in TNGL by a ranger. The ranger allowed them to continue without any repercussions. | 23. Seseorang yang tidak memiliki kekuasaan dari luar komunitas Anda ketahuan menebang pohon di TNGL oleh ranger. Ranger memperbolehkan mereka untuk melanjutkan tanpa konsekuensi. |
| 3 | 24 | 24. A powerful person from outside your community is caught logging in TNGL by a ranger. The ranger accepted a bribe and they were allowed to continue. | 24. Seseorang yang memiliki kekuasaan dari luar komunitas Anda ketahuan menebang pohon di TNGL oleh ranger. Ranger menerima sogokan mereka dan mereka diperbolehkan untuk melanjutkan. |
| 4 | 25 | 25. A powerful person from your community is caught logging in TNGL by a ranger. The ranger arrested them and they were prosecuted. | 25. Seseorang yang memiliki kekuasaan dari komunitas Anda ketahuan menebang pohon di TNGL oleh ranger. Ranger menangkap mereka dan mereka diproses hukum. |
| 4 | 26 | 26. A person with no power from outside your community is caught logging in TNGL by a ranger. The ranger accepted a bribe and they were allowed to continue. | 26. Seseorang yang tidak memiliki kekuasaan dari luar komunitas Anda ketahuan menebang pohon di TNGL oleh ranger. Ranger menerima sogokan mereka dan mereka diperbolehkan untuk melanjutkan. |
| 4 | 27 | 27. A powerful person from your community is caught hunting a protected sambar in TNGL by a ranger. The ranger accepted a bribe and they were allowed to continue. | 27. Seseorang yang memiliki kekuasaan dari komunitas Anda ketahuan berburu rusa yang dilindungi di TNGL oleh ranger. Ranger menerima sogokan dan mereka diperbolehkan untuk melanjutkan. |
| 4 | 28 | 28. A powerful person from outside your community is caught logging in TNGL by a ranger. The ranger confiscated the timber and warned them not to log again. | 28. Seseorang yang memiliki kekuasaan dari luar komunitas Anda ketahuan menebang pohon di TNGL oleh ranger. Ranger menyita barang bukti (contohnya: kayu dan gergaji mesin) dan memperingatkan mereka untuk tidak menebang lagi. |
| 4 | 29 | 29. A powerful person from outside your community is caught hunting a protected sambar in TNGL by a ranger. The ranger allowed them to continue without any repercussions. | 29. Seseorang yang memiliki kekuasaaan dari luar komunitas Anda ketahuan berburu rusa yang dilindungi di TNGL oleh ranger. Ranger memperbolehkan mereka untuk melanjutkan tanpa konsekuensi. |
| 4 | 30 | 30. A person with no power from your community is caught logging in TNGL by a ranger. The ranger allowed them to continue without any repercussions. | 30. Seseorang yang tidak memiliki kekuasaan dari komunitas Anda ketahuan menebang pohon di TNGL oleh ranger. Ranger memperbolehkan mereka untuk melanjutkan tanpa konsekuensi. |
| 4 | 31 | 31. A person with no power from outside your community is caught hunting a protected sambar in TNGL by a ranger. The ranger arrested them and they were prosecuted. | 31. Seseorang yang tidak memiliki kekuasaan dari luar komunitas Anda ketahuan berburu rusa yang dilindungi di TNGL oleh ranger. Ranger menangkap mereka dan mereka diproses hukum. |
| 4 | 32 | 32. A person with no power from your community is caught hunting a protected sambar in TNGL by a ranger. The ranger confiscated the sambar, and warned them not to hunt again. | 32. Seseorang yang tidak memiliki kekuasaan dari komunitas Anda ketahuan berburu rusa yang dilindungi di TNGL oleh ranger. Ranger menyita barang bukti (contohnya: rusa dan alat berburu) dan memperingatkan mereka untuk tidak berburu lagi. |

Table A1.4. Respondents in Indonesia were randomly allocated to one of the following vignettes in Experiment 2, which assessed the impact of corruption and norms on willingness to comply.

| **Vignette** | **Level of Compliance** | **Level of Corruption** | **English** | **Bahasa Indonesia** |
| --- | --- | --- | --- | --- |
| 1 | high | low | Members of your community rarely break rules by entering TNGL to collect resources. If caught breaking rules by a ranger, it is very likely that offenders will face criminal charges or fines. | Masyarakat di komunitas Anda jarang melanggar peraturan dengan memasuki TNGL untuk mengambil sumberdaya. Jika ketahuan melanggar peraturan oleh ranger, kemungkinan besar pelaku akan diproses hukum atau denda |
| 2 | low | high | Members of your community often break rules by entering TNGL to collect resources. If caught breaking rules by a ranger, it is very likely that offenders can pay a bribe and avoid any criminal charges. | Masyarakat di komunitas Anda sering melanggar peraturan dengan memasuki TNGL untuk mengambil sumberdaya. Jika ketahuan melanggar peraturan oleh ranger, kemungkinan besar pelaku dapat menyogok ranger dan terbebas dari semua proses hukum |
| 3 | high | high | Members of your community rarely break rules by entering TNGL to collect resources. If caught breaking rules by a ranger, it is very likely that offenders can pay a bribe and avoid any criminal charges. | Masyarakat di komunitas Anda jarang melanggar peraturan dengan memasuki TNGL untuk mengambil sumberdaya. Jika ketahuan melanggar peraturan oleh ranger, kemungkinan besar pelaku dapat menyogok ranger dan terbebas dari semua proses hukum |
| 4 | low | low | Members of your community often break rules by entering TNGL to collect resources. If caught breaking rules by a ranger, it is very likely that offenders will face criminal charges or fines. | Masyarakat di komunitas Anda sering melanggar peraturan dengan memasuki TNGL untuk mengambil sumberdaya. Jika ketahuan melanggar peraturan oleh ranger, kemungkinan besar pelaku akan diproses hukum atau denda |

Table A.1.5. Full vignette descriptions and their Kiswahili translations used in Experiment 1 – which assessed the perceived fairness of sanctions

| **Block** | **Vignette** | **English** | **Kiswahili** |
| --- | --- | --- | --- |
| 1 | 1 | 1. A powerful person from outside your community is caught grazing livestock in the Protected Area by a ranger. The ranger allowed them to continue without any repercussions. | 1. Mtu mwenye mamlaka kutoka nje ya jamii yako amekamatwa na askari wa wanyamapori akichunga mifugo ndani ya hifadhi. Askari wa wanyamapori akamruhusu kuendelea bila kumchukulia hatua yoyote. |
| 1 | 2 | 2. A person with no power from outside your community is caught hunting wildlife in the Protected Area by a ranger. The ranger accepted a bribe and they were allowed to continue. | 2. Mtu asiye na mamlaka kutoka nje ya jamii yako amekamatwa na askari wa wanyamapori akiwinda wanyamapori ndani ya hifadhi. Askari wa wanyamapori akapokea rushwa na akamruhusu kuendelea. |
| 1 | 3 | 3. A powerful person from outside your community is caught hunting wildlife in the Protected Area by a ranger. The ranger confiscated the wildlife, and warned them not to hunt again. | 3. Mtu mwenye mamlaka kutoka nje ya jamii yako amekamatwa na askari wa wanyamapori akiwinda wanyamapori ndani ya hifadhi. Askari wa wanyamapori akataifisha nyara na kumuonya asiwinde tena. |
| 1 | 4 | 4. A person with no power from your community is caught grazing livestock in the Protected Area by a ranger. The ranger confiscated the livestock and warned them not to graze there again. | 4. Mtu asiye na mamlaka kutoka kwenye jamii yako amekamatwa na askari wa wanyamapori akichunga mifugo ndani ya hifadhi. Askari wa wanyamapori akataifisha mifugo na kumuonya asichunge huko tena |
| 1 | 5 | 5. A powerful person from your community is caught hunting wildlife in the Protected Area by a ranger. The ranger arrested them and they were prosecuted. | 5. Mtu mwenye mamlaka kutoka kwenye jamii yako amekamatwa na askari wa wanyamapori akiwinda wanyamapori ndani ya hifadhi. Askari wa wanyamapori akamuweka chini ya ulinzi na kumfungulia mashtaka. |
| 1 | 6 | 6. A person with no power from outside your community is caught grazing livestock in the Protected Area by a ranger. The ranger arrested them and they were prosecuted. | 6. Mtu asiye na mamlaka kutoka nje ya jamii yako amekamatwa na askari wa wanyamapori akichunga mifugo ndani ya hifadhi. Askari wa wanyamapori akamuweka chini ya ulinzi na kumfungulia mashtaka. |
| 1 | 7 | 7. A person with no power from your community is caught hunting wildlife in the Protected Area by a ranger. The ranger allowed them to continue without any repercussions. | 7. Mtu asiye na mamlaka kutoka kwenye jamii yako amekamatwa na askari wa wanyamapori akiwinda wanyamapori ndani ya hifadhi. Askari wa wanyamapori akamruhusu kuendelea bila kumchukulia hatua yoyote. |
| 1 | 8 | 8. A powerful person from your community is caught grazing livestock in the Protected Area by a ranger. The ranger accepted a bribe and they were allowed to continue. | 8. Mtu mwenye mamlaka kutoka kwenye jamii yako amekamatwa na askari wa wanyamapori akichunga mifugo ndani ya hifadhi. Askari wa wanyamapori akapokea rushwa na akamruhusu kuendelea kuchunga. |
| 2 | 9 | 9. A person with no power from outside your community is caught hunting wildlife in the Protected Area by a ranger. The ranger allowed them to continue without any repercussions. | 9. Mtu asiye na mamlaka kutoka nje ya jamii yako amekamatwa na askari wa wanyamapori akiwinda wanyamapori ndani ya hifadhi. Askari wa wanyamapori akamruhusu kuendelea bila kumchukulia hatua yoyote. |
| 2 | 10 | 10. A powerful person from outside your community is caught grazing livestock in the Protected Area by a ranger. The ranger arrested them and they were prosecuted. | 10. Mtu mwenye mamlaka kutoka nje ya jamii yako amekamatwa na askari wa wanyamapori akichunga mifugo ndani ya hifadhi. Askari wa wanyamapori akamuweka chini ya ulinzi na kumfungulia mashtaka. |
| 2 | 11 | 11. A powerful person from your community is caught grazing livestock in the Protected Area by a ranger. The ranger allowed them to continue without any repercussions. | 11. Mtu mwenye mamlaka kutoka kwenye jamii yako amekamatwa na askari wa wanyamapori akichunga mifugo ndani ya hifadhi. Askari wa wanyamapori akamruhusu kuendelea bila kumchukulia hatua yoyote. |
| 2 | 12 | 12. A person with no power from outside your community is caught grazing livestock in the Protected Area by a ranger. The ranger confiscated the livestock and warned them not to graze there again. | 12. Mtu asiye na mamlaka kutoka nje ya jamii yako amekamatwa na askari wa wanyamapori akichunga mifugo ndani ya hifadhi. Askari wa wanyamapori akataifisha mifugo na kumuonya asichunge huko tena. |
| 2 | 13 | 13. A powerful person from outside your community is caught hunting wildlife in the Protected Area by a ranger. The ranger accepted a bribe and they were allowed to continue. | 13. Mtu mwenye mamlaka kutoka nje ya jamii yako amekamatwa na askari wa wanyamapori akiwinda wanyamapori ndani ya hifadhi. Askari wa wanyamapori akapokea rushwa na akamruhusu kuendelea. |
| 2 | 14 | 14. A powerful person from your community is caught hunting wildlife in the Protected Area by a ranger. The ranger confiscated the wildlife, and warned them not to hunt again. | 14. Mtu mwenye mamlaka kutoka kwenye jamii yako amekamatwa na askari wa wanyamapori akiwinda wanyamapori ndani ya hifadhi. Askari wa wanyamapori akataifisha nyara na kumuonya asiwinde tena. |
| 2 | 15 | 15. A person with no power from your community is caught hunting wildlife in the Protected Area by a ranger. The ranger arrested them and they were prosecuted. | 15. Mtu asiye na mamlaka kutoka kwenye jamii yako amekamatwa na askari wa wanyamapori akiwinda wanyamapori ndani ya hifadhi. Askari wa wanyamapori akamuweka chini ya ulinzi na kumfungulia mashtaka. |
| 2 | 16 | 16. A person with no power from your community is caught grazing livestock in the Protected Area by a ranger. The ranger accepted a bribe and they were allowed to continue. | 16. Mtu asiye na mamlaka kutoka kwenye jamii yako amekamatwa na askari wa wanyamapori akichunga mifugo ndani ya hifadhi. Askari wa wanyamapori akapokea rushwa na akamruhusu kuendelea kuchunga. |
| 3 | 17 | 17. A person with no power from outside your community is caught hunting wildlife in the Protected Area by a ranger. The ranger confiscated the wildlife, and warned them not to hunt again. | 17. Mtu asiye na mamlaka kutoka nje ya jamii yako amekamatwa na askari wa wanyamapori akiwinda wanyamapori ndani ya hifadhi. Askari wa wanyamapori akataifisha nyara na kumuonya asiwinde tena. |
| 3 | 18 | 18. A powerful person from outside your community is caught hunting wildlife in the Protected Area by a ranger. The ranger arrested them and they were prosecuted. | 18. Mtu mwenye mamlaka kutoka nje ya jamii yako amekamatwa na askari wa wanyamapori akiwinda wanyamapori ndani ya hifadhi. Askari wa wanyamapori akamuweka chini ya ulinzi na kumfungulia mashtaka. |
| 3 | 19 | 19. A powerful person from your community is caught grazing livestock in the Protected Area by a ranger. The ranger confiscated the livestock and warned them not to graze there again. | 19. Mtu mwenye mamlaka kutoka kwenye jamii yako amekamatwa na askari wa wanyamapori akichunga mifugo ndani ya hifadhi. Askari wa wanyamapori akataifisha mifugo na kumuonya asichunge huko tena. |
| 3 | 20 | 20. A person with no power from your community is caught grazing livestock in the Protected Area by a ranger. The ranger arrested them and they were prosecuted. | 20. Mtu asiye na mamlaka kutoka kwenye jamii yako amekamatwa na askari wa wanyamapori akichunga mifugo ndani ya hifadhi. Askari wa wanyamapori akamuweka chini ya ulinzi na kumfungulia mashtaka. |
| 3 | 21 | 21. A powerful person from your community is caught hunting wildlife in the Protected Area by a ranger. The ranger allowed them to continue without any repercussions. | 21. Mtu mwenye mamlaka kutoka kwenye jamii yako amekamatwa na askari wa wanyamapori akiwinda wanyamapori ndani ya hifadhi. Askari wa wanyamapori akamruhusu kuendelea bila kumchukulia hatua yoyote. |
| 3 | 22 | 22. A person with no power from your community is caught hunting wildlife in the Protected Area by a ranger. The ranger accepted a bribe and they were allowed to continue. | 22. Mtu asiye na mamlaka kutoka kwenye jamii yako amekamatwa na askari wa wanyamapori akiwinda wanyamapori ndani ya hifadhi. Askari wa wanyamapori akapokea rushwa na akamruhusu kuendelea. |
| 3 | 23 | 23. A person with no power from outside your community is caught grazing livestock in the Protected Area by a ranger. The ranger allowed them to continue without any repercussions. | 23. Mtu asiye na mamlaka kutoka nje ya jamii yako amekamatwa na askari wa wanyamapori akichunga mifugo ndani ya hifadhi. Askari wa wanyamapori akamruhusu kuendelea bila kuchukulia hatua yoyote. |
| 3 | 24 | 24. A powerful person from outside your community is caught grazing livestock in the Protected Areaby a ranger. The ranger accepted a bribe and they were allowed to continue. | 24. Mtu mwenye mamlaka kutoka nje ya jamii yako amekamatwa na askari wa wanyamapori akichunga mifugo ndani ya hifadhi. Askari wa wanyamapori akapokea rushwa na akamruhusu kuendelea kuchunga. |
| 4 | 25 | 25. A powerful person from your community is caught grazing livestock in the Protected Area by a ranger. The ranger arrested them and they were prosecuted. | 25. Mtu mwenye mamlaka kutoka kwenye jamii yako amekamatwa na askari wa wanyamapori akichunga mifugo ndani ya hifadhi. Askari wa wanyamapori akamuweka chini ya ulinzi na kumfungulia mashtaka. |
| 4 | 26 | 26. A person with no power from outside your community is caught grazing livestock in the Protected Area by a ranger. The ranger accepted a bribe and they were allowed to continue. | 26. Mtu asiye na mamlaka kutoka nje ya jamii yako amekamatwa na askari wa wanyamapori akichunga mifugo ndani ya hifadhi. Askari wa wanyamapori akapokea rushwa na akamruhusu kuendelea kuchunga. |
| 4 | 27 | 27. A powerful person from your community is caught hunting wildlife in the Protected Area by a ranger. The ranger accepted a bribe and they were allowed to continue. | 27. Mtu mwenye mamlaka kutoka kwenye jamii yako amekamatwa na askari wa wanyamapori akiwinda wanyamapori ndani ya hifadhi. Askari wa wanyamapori akapokea rushwa na akamruhusu kuendelea. |
| 4 | 28 | 28. A powerful person from outside your community is caught grazing livestock in the Protected Area by a ranger. The ranger confiscated the livestock and warned them not to graze there again. | 28. Mtu mwenye mamlaka kutoka nje ya jamii yako amekamatwa na askari wa wanyamapori akichunga mifugo ndani ya hifadhi. Askari wa wanyamapori akataifisha mifugo na kumuonya asichunge huko tena. |
| 4 | 29 | 29. A powerful person from outside your community is caught hunting wildlife in the Protected Area by a ranger. The ranger allowed them to continue without any repercussions. | 29. Mtu mwenye mamlaka kutoka nje ya jamii yako amekamatwa na askari wa wanyamapori akiwinda wanyamapori ndani ya hifadhi. Askari wa wanyamapori akamruhusu kuendelea bila kumchukulia hatua yoyote. |
| 4 | 30 | 30. A person with no power from your community is caught grazing livestock in the Protected Areaby a ranger. The ranger allowed them to continue without any repercussions. | 30. Mtu asiye na mamlaka kutoka kwenye jamii yako amekamatwa na askari wa wanyamapori akichunga mifugo ndani ya hifadhi. Askari wa wanyamapori akamruhusu kuendelea bila kumchukulia hatua yoyote. |
| 4 | 31 | 31. A person with no power from outside your community is caught hunting wildlife in the Protected Area by a ranger. The ranger arrested them and they were prosecuted. | 31. Mtu asiye na mamlaka kutoka nje ya jamii yako amekamatwa na askari wa wanyamapori akiwinda wanyamapori ndani ya hifadhi. Askari wa wanyamapori akamuweka chini ya ulinzi na kumfungulia mashtaka. |
| 4 | 32 | 32. A person with no power from your community is caught hunting wildlife in the Protected Area by a ranger. The ranger confiscated the wildlife, and warned them not to hunt again. | 32. Mtu asiye na mamlaka kutoka kwenye jamii yako amekamatwa na askari wa wanyamapori akiwinda wanyamapori ndani ya hifadhi. Askari wa wanyamapori akataifisha nyara na kumuonya asiwinde tena. |

Table A1.6. Respondents in Tanzania were randomly allocated to one of the following vignettes in Experiment 2, which assessed the impact of corruption and norms on willingness to comply.

| **Vignette** | **Compliance** | **Corruption** | **English** | **Kiswahili** |
| --- | --- | --- | --- | --- |
| 1 | high | low | Members of your community rarely break rules by entering the Protected Area to collect resources. If caught breaking rules by a ranger, it is very likely that offenders will face criminal charges or fines. | Wanajamii wa jamii yenu huvunja sheria mara chache kwa kuingia ndani ya Hifadhi kukusanya rasilimali. Kama wakikamatwa na askari wa wanyamapori wakivunja sheria, kuna uwezekano mkubwa kwamba wakosaji watakabiliwa na mashtaka ya jinai au faini. |
| 2 | low | high | Members of your community often break rules by entering the Protected Area to collect resources. If caught breaking rules by a ranger, it is very likely that offenders can pay a bribe and avoid any criminal charges. | Wanajamii wa jamii yenu huvunja sheria mara nyingi kwa kuingia ndani ya Hifadhi kukusanya rasilimali. Kama wakikamatwa na askari wa wanyamapori wakivunja sheria, kuna uwezekano mkubwa kwamba wakosaji wanaweza kutoa rushwa na kuepuka mashtaka yoyote ya jinai. |
| 3 | high | high | Members of your community rarely break rules by entering the Protected Area to collect resources. If caught breaking rules by a ranger, it is very likely that offenders can pay a bribe and avoid any criminal charges. | Wanajamii wa jamii yenu huvunja sheria mara chache kwa kuingia ndani ya Hifadhi kukusanya rasilimali. Kama wakikamatwa na askari wa wanyamapori wakivunja sheria, kuna uwezekano mkubwa kwamba wakosaji wanaweza kutoa rushwa na kuepuka mashtaka yoyote ya jinai. |
| 4 | low | low | Members of your community often break rules by entering the Protected Area to collect resources. If caught breaking rules by a ranger, it is very likely that offenders will face criminal charges or fines. | Wanajamii wa jamii yenu huvunja sheria mara nyingi kwa kuingia ndani ya Hifadhi kukusanya rasilimali. Kama wakikamatwa na askari wa wanyamapori wakivunja sheria, kuna uwezekano mkubwa kwamba wakosaji watakabiliwa na mashtaka ya jinai au faini. |

### Indonesian Survey Instrument

Table A1.7. Survey instrument in English and in Bahasa Indonesia.

| **Question (English)** | **Question (Bahasa Indonesia)** | **Answer options (English)** | **Answer options (Bahasa Indonesia)** |
| --- | --- | --- | --- |
| Section 1: Survey location (interviewer completes) | | | |
| Province | Provinsi |  |  |
| District | Kabupaten |  |  |
| What is the district? | Kabupaten apa? |  |  |
| Sub-district | Kecamatan |  |  |
| Village | Desa |  |  |
| Sub-village | Dusun |  |  |
| Who is conducting the interview? | Siapa yang melakukan wawancara? |  |  |
| What gender is the respondent? | Apa jenis kelamin responden? |  |  |
| Section 2: Experimental design details (not shown to respondent) | | | |
| Vignette order: ${v_order} | Vignette order: ${v_order} |  |  |
| Vignettes selected from block: ${block} | Vignette dipilih dari blok: ${block} |  |  |
| Scenario number: ${rand_scenario} | Scenario number: ${rand_scenario} |  |  |
| Section 3: Consent | | | |
| My name is ${interviewer} and I work for a project being conducted by Bangor University in the United Kingdom. Today, I would like to invite you to take part in a short study.   The aim of the study is to understand what life is like for people like you that live close to a protected area. I am asking you to take part because you live close to a one. In total we are hoping to speak to between 200 and 300 people like you who live close to a protected area.   If you agree to take part, I will ask you some questions and record your answer on this device [show questionnaire on tablet]. I will never record your name or your address on this, or any other form; your answers are private and confidential. The first set of questions is about you and your household, then I will ask about your opinions about the rules of the protected area. This should take between 30 minutes and one hour.   Because I am not recording your name, your answers cannot be attributed to you, and it will not be possible to identify you from our records. Information you provide will not be shared with anybody outside of the research team and will only be used by members of the research team based in the UK. Your answers, together with those of other respondents, will be used to write reports.   The study has been approved by the Bangor University Ethics Committee. If you would prefer not to take part, that is ok. Also, if you agree at first but then change your mind and would like to stop at any point, please tell me and I will stop immediately. Before going any further, do you have any questions that you would like to ask me?   If you have any concerns please contact us on this phone number [provide contact card].   Would you like to take part in this study? | Nama saya ${interviewer} dan saya bekerja untuk proyek penelitian yang dilakukan oleh Universitas Bangor di Inggris. Hari ini, saya akan mengajak Anda untuk ikut serta dalam studi yang pendek.   Maksud dari penelitian ini adalah untuk memahami seperti apa kehidupan masyarakat seperti Bapak/Ibu yang tinggal di dekat kawasan lindung. Saya mengajak Bapak/Ibu untuk ikut serta karena Bapak/Ibu tinggal berdekatan dengan salah satu kawasan. Secara keseluruhan, kami berharap bisa bertanya pada sekitar 200 sampai 300 orang seperti Bapak/Ibu yang tinggal di dekat kawasan lindung.   Jika Bapak/Ibu bersedia untuk ikut serta, saya akan menanyakan beberapa pertanyaan dan mencatat jawaban Bapak/Ibu di alat ini [tunjukkan kuesioner di tablet]. Saya tidak akan pernah mencatat nama atau alamat Bapak/Ibu di sini, atau juga di lembar yang lain; jawaban Bapak/Ibu bersifat pribadi dan rahasia. Bagian pertama pertanyaan adalah tentang Bapak/Ibu dan rumah tangga Bapak/Ibu, kemudian saya akan bertanya tentang opini Bapak/Ibu mengenai peraturan-peraturan dari kawasan lindung. Ini mungkin akan memakan waktu sekitar 30 menit sampai 1 jam.   Karena saya tidak mencatat nama Bapak/Ibu, jawaban Bapak/Ibu tidak dapat dikaitkan dengan Bapak/Ibu, dan juga tidak mungkin untuk mengidentifikasikan Bapak/Ibu dari catatan kami. Informasi yang Bapak/Ibu berikan tidak akan dibagikan kepada siapapun di luar tim penelitian dan hanya akan digunakan oleh anggota tim penelitian yang berbasis di Inggris. Jawaban Bapak/Ibu, bersama-sama dengan responden lainnya, akan digunakan untuk menulis laporan penelitian.   Penelitian ini telah disetujui oleh Komite Etik Universitas Bangor. Jika Bapak/Ibu memilih untuk tidak ikut serta, silakan saja. Juga, jika Bapak/Ibu awalnya setuju tapi kemudian berubah pikiran dan ingin berhenti di bagian tertentu, mohon katakan pada saya dan saya akan langsung berhenti. Sebelum lebih lanjut, apakah Bapak/Ibu memiliki pertanyaan yang ingin ditanyakan kepada saya?   Jika Bapak/Ibu memiliki kekhawatiran atau sesuatu yang mengganjal, silakan hubungi kami melalui nomor ini [berikan kartu kontak].   Apakah Bapak/Ibu bersedia untuk ikut serta dalam penelitian ini? | Yes / No | Iya / Tidak |
| The respondent must agree to the following statements:   • I confirm that I have had the Information Sheet read out to me and I understand the information provided for this study. I have had the opportunity to ask questions if necessary and have had these answered satisfactorily.  • I understand that my participation is voluntary and that I am free to withdraw at any time without giving any reason. If I withdraw my data will be removed from the study and will be destroyed.  • I understand that the answers I provide will be used for the purposes explained to me.  • Based upon the above, I agree to take part in this study. | Responden harus menyetujui pernyataan berikut:   **Saya menyatakan bahwa saya telah mendapatkan lembar Informasi ConHub yang dibacakan kepada saya dan saya mengerti informasi terkait penelitian ini. Saya memiliki kesempatan untuk bertanya jika perlu dan sudah mendapatkan jawaban yang memuaskan**   **Saya mengerti bahwa keikutsertaan saya bersifat sukarela dan bahwa saya bebas untuk tidak melanjutkan kapan saja tanpa memberikan alasan tertentu. Jika saya tidak melanjutkan, data saya akan dibuang dari penelitian ini dan dihilangkan. **  **Saya mengerti bahwa jawaban yang saya berikan akan digunakan sesuai yang sudah dijelaskan kepada saya.**   **Berdasarkan hal tersebut, saya bersedia untuk ikut serta dalam penelitian ini. ** " | Yes / No | Iya / Tidak |
| Section 4: COVID-19 precautions | | | |
| If the guide has not already explained our COVID precautions then explain to the respondent:  1. That the team are clear of symptoms  2. That the team are taking regular covid tests before starting fieldwork.  3. That we wear masks to protect ourselves and the respondent  4. That we will be working outside and maintaining social distancing  5. We will be washing hands frequently | Jika pemandu/pendamping belum menjelaskan tentang tindakan pencegahan COVID yang sudah dilakukan oleh Tim, jelaskan kepada responden:  1. Bahwa tim bebas dari gejala apapun  2. Bahwa tim sudah melakukan cek rutin COVID sebelum ke lapangan 3. Bahwa tim menggunakan masker untuk melindungi diri sendiri dan responden  4. Bahwa tim akan melakukan kegiatan di luar atau area terbuka dan menjaga jarak  5. Tim akan rutin mencuci tangan |  |  |
| Does anyone in your household have symptoms of COVID19 that have developed over the previous week?   These are:  * a new and persistent cough, * difficulty in breathing, * a high fever,  * a recent loss of taste or smell? | Apakah ada orang di rumah tangga Anda yang memiliki gejala COVID19 dalam seminggu terakhir?  Gejalanya adalah:  * batuk yang baru terjadi dan terus-menerus, * kesulitan bernafas, * demam tinggi,  * dan kehilangan kemampuan pengecapan atau penciuman? |  |  |
| Thank the respondent for their time, explain that even though the sick individual may not have COVID we do not want to put other respondents at risk if they do have COVID. Wish them or their household member a quick recovery | Berterima kasih kepada responden atas waktu mereka, jelaskan meskipun orang yang sakit tidak menderita COVID, kami tidak mau menyebabkan responden lain beresiko menderita COVID. Doakan semoga responden atau anggota keluaga mereka cepat pulih. |  |  |
| Section 5: Respondent demographics | | | |
| I am going to ask you a few questions about yourself and the others in your household, by household I mean those who eat together and sleep in the same house, including children who may be at boarding school | Saya akan mengajukan pertanyaan tentang diri Anda dan anggota rumah tangga Anda, rumah tangga yang saya maksud adalah mereka yang makan dan tidur di rumah yang sama, termasuk anak-anak yang mungkin tinggal di asrama/bersekolah di luar |  |  |
| 1. How old are you? | 1. Berapa usia Anda? |  |  |
| 2. How many people are there in your household? | 2. Berapa banyak orang yang tinggal di rumah tangga Anda? |  |  |
| 3a. What is the ethnic majority of your household? | 3a. Apa etnis terbesar di rumah tangga Anda? |  |  |
| 3b. Please specify which ethnic group | 3b. Tolong jelaskan etnik yang mana |  |  |
| 4. How many years have you lived in this village? | 4. Sudah berapa lama Anda tinggal di desa ini? |  |  |
| 5. Are you currently attending school? | 5. Apakah saat ini Anda masih bersekolah? |  |  |
| 6. How many years of schooling have you completed? | 6. Berapa tahun pendidikan yang pernah Anda tempuh? |  |  |
| 7. How many members of the household who are aged 12 years or older, have completed at least 5 years of schooling? | 7. Ada berapa anggota rumah tangga yang berusia 12 tahun atau diatas yang menyelesaikan sekolah minimal 5 tahun? |  |  |
| 8. Are there any children in the household aged between 7 and 18 who do not go to school? | 8. Apakah ada anak berusia 7 sampai 18 tahun di rumah tangga anda yang tidak bersekolah? |  |  |
| Section 6: Multi-dimensional Poverty Assessment | | | |
| 1. What is your households main livelihood activity? | 1. Bisakah Anda memberitahu daftar mata pencaharian anggota rumah tangga Anda |  |  |
| 2. Does the household's main residence have electricity? | 2. Apakah rumah tinggal utama rumah tangga memiliki listrik? |  |  |
| 3. Does the household have access to a toilet? | 3. Apakah rumah tangga punya akses ke toilet? |  |  |
| 3a. What type of toilet is this? | 3a. Apa jenis toiletnya? |  |  |
| 3b. What is the 'other' type of toilet? | 3b. Apa jenis toilet yang 'lain'? |  |  |
| 4. Does the household share this toilet with other households? | 4. Apakah rumah tangga ini berbagi toilet dengan rumah tangga lain? |  |  |
| 5a. What is the household's main source of drinking water? | 5a. Apa sumber utama air minum dalam rumah tangga ini? |  |  |
| 5b. What is the 'other' water source? | 5b. Apa jenis sumber air 'lain'? |  |  |
| 6. How many minutes does it take to travel to the household's main source of drinking water? There and back using the mode of transport you use most often to collect water | 6. Berapa menit jarak yang ditempuh rumah tangga ke sumber air minum? Pulang pergi dengan menggunakan transportasi yang paling sering digunakan untuk mengambil air |  |  |
| 7a. What is the floor of the household's main residence made from? | 7a. Terbuat dari apa lantai dari tempat tinggal utama rumah tangga ini? |  |  |
| 7b. What is the 'other' floor material? | 7b. Terbuat dari bahan apa lantai yang 'lain'? |  |  |
| 8a. What is the household's main cooking fuel? | 8a. Apa bahan bakar utama memasak yang digunakan rumah tangga? |  |  |
| 8b. What is the 'other' cooking fuel? | 8b. Apa bahan bakar 'lain'? |  |  |
| 9. Has a child under the age of 18 died in the household in the last 5 years? | 9. Apakah ada anak usia di bawah 18 tahun dalam rumah tangga ini yang meninggal dalam 5 tahun terkahir? |  |  |
| 10. How many children under the age of 18 have died in the household in the last 5 years? | 10. Berapa banyak anak usia di bawah 18 tahun yang meninggal di rumah tangga ini dalam 5 tahun terkahir? |  |  |
| I am now going to ask about your households’ access to food over the last 12 months" | Saya akan mengajukan pertanyaan tentang bagaimana cara rumah tangga Anda memenuhi kebutuhan pangan dalam 12 bulan terakhir |  |  |
| 11. In the past 12 months, how often was there no food to eat of any kind in your house because of lack of resources to get food? | 11. Dalam 12 bulan terakhir, seberapa sering ketiadaan makanan dalam rumah tangga Anda karena kurangnya sumber daya untuk mendapatkan makanan? |  |  |
| 12. In the past 12 months, how often did any household member go to sleep at night hungry because there was not enough food? | 12. Dalam 12 bulan terakhir, seberapa sering anggota rumah tangga tidur malam hari dalam keadaan lapar karena tidak ada cukup makanan? |  |  |
| 13. In the past 12 months, how often did any household member go a whole day and night without eating anything because there was not enough food? | 13. Dalam 12 bulan terkahir, seberapa sering anggota rumah tangga tidak makan apapun sehari semalam karena tidak ada cukup makanan? |  |  |
| The following questions are about the items that you and your household own. | Pertanyaan berikut adalah tentang barang-barang yang Anda dan rumah tangga Anda miliki |  |  |
| 14. How many radios does the household own? | 14. Berapa banyak radio yang dimiliki rumah tangga Anda? |  |  |
| 15. How many TVs does the household own? | 15. Berapa banyak TV yang dimiliki rumah tangga Anda? |  |  |
| 16. How many mobile phones does the household own? | 16. Berapa banyak telepon genggam yang dimiliki rumah tangga Anda? |  |  |
| 17. How many bicycles does the household own? | 17. Berapa banyak sepeda yang dimiliki rumah tangga Anda? |  |  |
| 18. How many motorbikes does the household own? | 18. Berapa banyak sepeda motor yang dimiliki rumah tangga Anda? |  |  |
| 19. How many refrigerators does the household own? | 19. Berapa banyak kulkas yang dimiliki rumah tangga Anda? |  |  |
| 20. How many cars or trucks does the household own? | 20. Berapa banyak mobil atau truk yang dimiliki rumah tangga Anda? |  |  |
| The next questions are about how you perceive yourself and your household. Please answer on a scale of strongly agree to strongly disagree | Pertanyaan berikut adalah tentang bagaimana Anda memandang diri Anda dan rumah tangga Anda. Tolong jawab dalam skala sangat setuju ke sangat tidak setuju. |  |  |
| 21. Your household is poor | 21. Rumah tangga Anda miskin | Strongly agree, Agree, Neither agree or disagree, Disagree, Strongly disagree, Prefer not to answer, Don’t know | Sangat setuju, Setuju, Netral, Tidak setuju, Sangat tidak setuju, Memilih untuk tidak menjawab, Tidak tahu |
| 22. Your household is happy | 22. Rumah tangga Anda bahagia |  |  |
| 23. Yours is a prosperous household | 23. Rumah tangga Anda sejahtera |  |  |
| 24. Community members are willing to help each other (e.g., with work, food or financially) | 24. Anggota masyarakat bersedia untuk membantu satu sama lain (misalnya, terkait pekerjaan, makanan atau keuangan) |  |  |
| 25. Trust among community members in this village is strong | 25. Tingkat kepercayaan di masyarakat ini tinggi |  |  |
| 26. Conflicts frequently arise between people or families in this community | 26. Konflik sering muncul antara orang dan keluarga di masyarakat ini |  |  |
| 27. The amount of land your household has at the moment means you are prosperous | 27. Luas tanah yang anda miliki saat ini menandakan bahwa anda makmur |  |  |
| 28. ${interviewer}, any comments on the poverty section? | 28. ${interviewer}, ada komentar di Bagian Kemiskinan (poverty)? |  |  |
| Section 7: Factorial Survey Experiment 🡪 ${v_X_q} – represents the vignette number shown to the respondent | | | |
| I will now read you several short descriptions of hypothetical interactions between rangers of TNGL and people who enter the National Park. For each one, I would like you to consider the situation, and tell me how fair your think the outcome is. | Sekarang Saya akan membacakan beberapa deskripsi singkat dari interaksi yang mungkin terjadi antara ranger TNGL dan masyarakat yang memasuki Taman Nasional. Untuk masing-masing deskripsi, Saya ingin Bapak/Ibu memikirkan situasinya, dan beritahu Saya seberapa adil menurut Bapak/Ibu hasilnya |  |  |
| Vignette 1 : ${v_1_q} How fair do you think this? | Vignette 1 : ${v_1_q} Seberapa adil menurut Anda | Very fair, Fair, Neither fair nor unfair, Unfair, Very unfair, Prefer not to answer, Don’t know | Dengan sangat adil, Dengan adil, Netral, Dengan tidak adil, Dengan sangat tidak adil, Memilih untuk tidak menjawab, Tidak tahu |
| v1b. How likely do you think it is that this situation would happen in real life? | v1b. Seberapa mungkin menurut Anda situasi tersebut akan terjadi di kehidupan nyata? | Very likely, Likely, Neither likely nor unlikely, Unlikely, Very unlikely, Prefer not to answer, Don’t know | Sangat mungkin, Mungkin, Netral, Tidak mungkin, Sangat tidak mungkin, Memilih untuk tidak menjawab, Tidak tahu |
| Vignette 2 : ${v_2_q} How fair do you think this? | Vignette 2 : ${v_2_q} Seberapa adil menurut Anda | Very fair, Fair, Neither fair nor unfair, Unfair, Very unfair, Prefer not to answer, Don’t know | Dengan sangat adil, Dengan adil, Netral, Dengan tidak adil, Dengan sangat tidak adil, Memilih untuk tidak menjawab, Tidak tahu |
| v2b. How likely do you think it is that this situation would happen in real life? | v2b. Seberapa mungkin menurut Anda situasi tersebut akan terjadi di kehidupan nyata? | Very likely, Likely, Neither likely nor unlikely, Unlikely, Very unlikely, Prefer not to answer, Don’t know | Sangat mungkin, Mungkin, Netral, Tidak mungkin, Sangat tidak mungkin, Memilih untuk tidak menjawab, Tidak tahu |
| Vignette 3 : ${v_3_q} How fair do you think this? | Vignette 3 : ${v_3_q} Seberapa adil menurut Anda | Very fair, Fair, Neither fair nor unfair, Unfair, Very unfair, Prefer not to answer, Don’t know | Dengan sangat adil, Dengan adil, Netral, Dengan tidak adil, Dengan sangat tidak adil, Memilih untuk tidak menjawab, Tidak tahu |
| v3b. How likely do you think it is that this situation would happen in real life? | v3b. Seberapa mungkin menurut Anda situasi tersebut akan terjadi di kehidupan nyata? | Very likely, Likely, Neither likely nor unlikely, Unlikely, Very unlikely, Prefer not to answer, Don’t know | Sangat mungkin, Mungkin, Netral, Tidak mungkin, Sangat tidak mungkin, Memilih untuk tidak menjawab, Tidak tahu |
| Vignette 4 : ${v_4_q} How fair do you think this? | Vignette 4 : ${v_4_q} Seberapa adil menurut Anda | Very fair, Fair, Neither fair nor unfair, Unfair, Very unfair, Prefer not to answer, Don’t know | Dengan sangat adil, Dengan adil, Netral, Dengan tidak adil, Dengan sangat tidak adil, Memilih untuk tidak menjawab, Tidak tahu |
| v4b. How likely do you think it is that this situation would happen in real life? | v4b. Seberapa mungkin menurut Anda situasi tersebut akan terjadi di kehidupan nyata? | Very likely, Likely, Neither likely nor unlikely, Unlikely, Very unlikely, Prefer not to answer, Don’t know | Sangat mungkin, Mungkin, Netral, Tidak mungkin, Sangat tidak mungkin, Memilih untuk tidak menjawab, Tidak tahu |
| Vignette 5 : ${v_5_q} How fair do you think this? | Vignette 5 : ${v_5_q} Seberapa adil menurut Anda | Very fair, Fair, Neither fair nor unfair, Unfair, Very unfair, Prefer not to answer, Don’t know | Dengan sangat adil, Dengan adil, Netral, Dengan tidak adil, Dengan sangat tidak adil, Memilih untuk tidak menjawab, Tidak tahu |
| v5b. How likely do you think it is that this situation would happen in real life? | v5b. Seberapa mungkin menurut Anda situasi tersebut akan terjadi di kehidupan nyata? | Very likely, Likely, Neither likely nor unlikely, Unlikely, Very unlikely, Prefer not to answer, Don’t know | Sangat mungkin, Mungkin, Netral, Tidak mungkin, Sangat tidak mungkin, Memilih untuk tidak menjawab, Tidak tahu |
| Vignette 6 : ${v_6_q} How fair do you think this? | Vignette 6 : ${v_6_q} Seberapa adil menurut Anda | Very fair, Fair, Neither fair nor unfair, Unfair, Very unfair, Prefer not to answer, Don’t know | Dengan sangat adil, Dengan adil, Netral, Dengan tidak adil, Dengan sangat tidak adil, Memilih untuk tidak menjawab, Tidak tahu |
| v6b. How likely do you think it is that this situation would happen in real life? | v6b. Seberapa mungkin menurut Anda situasi tersebut akan terjadi di kehidupan nyata? | Very likely, Likely, Neither likely nor unlikely, Unlikely, Very unlikely, Prefer not to answer, Don’t know | Sangat mungkin, Mungkin, Netral, Tidak mungkin, Sangat tidak mungkin, Memilih untuk tidak menjawab, Tidak tahu |
| Vignette 7 : ${v_7_q} How fair do you think this? | Vignette 7 : ${v_7_q} Seberapa adil menurut Anda | Very fair, Fair, Neither fair nor unfair, Unfair, Very unfair, Prefer not to answer, Don’t know | Dengan sangat adil, Dengan adil, Netral, Dengan tidak adil, Dengan sangat tidak adil, Memilih untuk tidak menjawab, Tidak tahu |
| v7b. How likely do you think it is that this situation would happen in real life? | v7b. Seberapa mungkin menurut Anda situasi tersebut akan terjadi di kehidupan nyata? | Very likely, Likely, Neither likely nor unlikely, Unlikely, Very unlikely, Prefer not to answer, Don’t know | Sangat mungkin, Mungkin, Netral, Tidak mungkin, Sangat tidak mungkin, Memilih untuk tidak menjawab, Tidak tahu |
| Vignette 8 : ${v_8_q} How fair do you think this? | Vignette 8 : ${v_8_q} Seberapa adil menurut Anda | Very fair, Fair, Neither fair nor unfair, Unfair, Very unfair, Prefer not to answer, Don’t know | Dengan sangat adil, Dengan adil, Netral, Dengan tidak adil, Dengan sangat tidak adil, Memilih untuk tidak menjawab, Tidak tahu |
| v8b. How likely do you think it is that this situation would happen in real life? | v8b. Seberapa mungkin menurut Anda situasi tersebut akan terjadi di kehidupan nyata? | Very likely, Likely, Neither likely nor unlikely, Unlikely, Very unlikely, Prefer not to answer, Don’t know | Sangat mungkin, Mungkin, Netral, Tidak mungkin, Sangat tidak mungkin, Memilih untuk tidak menjawab, Tidak tahu |
| Section 8: Scenarios | | | |
| Please tell me to what extent you agree or disagree with the following statements | Tolong beritahu Saya sejauh mana Anda setuju atau tidak setuju dengan pernyataan berikut |  |  |
| 1. Violating TNGL rules risks the good name of people in the community | 1. Melanggar peraturan TNGL membahayakan nama baik masyarakat di desa/komunitas ini | Strongly agree, Agree, Neither agree or disagree, Disagree, Strongly disagree, Prefer not to answer, Don’t know | Sangat setuju, Setuju, Netral, Tidak setuju, Sangat tidak setuju, Memilih untuk tidak menjawab, Tidak tahu |
| 2. The rules of TNGL support conservation of biodiversity | 2. Peraturan TNGL mendukung konservasi keanekaragaman hayati |  |  |
| 3. The rules of TNGL are fair and consistent with the law | 3. Peraturan TNGL adil dan sejalan dengan peraturan/hukum |  |  |
| 4. Breaking rules is considered a disloyal towards fellow members of the community | 4. Melanggar peraturan dianggap tidak setia terhadap sesama masyarakat di desa/komunitas |  |  |
| I will now read you out an imaginary scenario. Based on this scenario I will ask you some questions, please answer them as honestly as you can. | Sekarang Saya akan membacakan skenario khayalan. Berdasarkan skenario ini, Saya akan menanyakan beberapa pertanyaan, tolong dijawab secara jujur |  |  |
| Imagine the following situation. ${scenario} | Bayangkan situasi berikut. ${scenario} |  |  |
| 5. If this scenario were true, how willing would you be, in general to follow the rules of TNGL? | 5. Jika skenario semacam ini benar terjadi, seberapa bersedia Anda, secara umum mengikuti peraturan TNGL? | Very willing, Wiling, neither willing nor unwilling, Unwilling, Very unwilling, Prefer not to answer, Don’t know | Sangat bersedia, Bersedia, Netral, Tidak bersedia, Sangat tidak bersedia, Memilih untuk tidak menjawab, Tidak tahu |
| 6. If this scenario were true, how often in the coming months would you follow the rules of TNGL? | 6. Jika skenario semacam ini benar terjadi, seberapa sering dalam beberapa bulan mendatang Anda akan mengikuti peraturan TNGL? | Every Time, Almost every time, Occasionally, Rarely, Never, Prefer not to answer, Don’t know | Setiap saat, Hampir setiap saat, Kadang-kadang, Jarang, Tidak pernah, Memilih untuk tidak menjawab, Tidak tahu |
| 7. If this scenario were true, to what extent would you consider violating the rules of TNGL? | 7. Jika skenario semacam ini benar terjadi, sejauh mana Anda mempertimbangkan untuk melanggar peraturan TNGL? | Definitely consider, May or may not consider, Would definitely not consider, Prefer not to answer, Don’t know | Sangat mempertimbangkan, Mungkin mempertimbangkan, Tidak akan mempertimbangkan, Memilih untuk tidak menjawab, Tidak tahu |
| Section 9: End of survey | | | |
| The survey is now finished. Thank you for participating. Do you have any questions for me? | Survey ini sudah selesai. Terimakasih sudah berpartisipasi. Apakah anda memiliki pertanyaan untuk saya? |  |  |
| ${interviewer}, how engaged was the participant throughout the survey? | ${interviewer}, seberapa besar partisipasi peserta selama wawancara berlangsung? | Very engaged, Engaged, Neutral, Unengaged, Very unengaged | Sangat tertarik, Tertarik, Netral, Tidak tertarik, Sangat tidak tertarik |
| ${interviewer}, how did you find surveying this individual? | ${interviewer}, bagaimana pendapatmu melakukan survey pada orang ini? | Very easy, easy, Neutral, Hard, Very hard | Sangat mudah, Cukup mudah, Tidak mudah tapi juga tidak sulit, Cukup sulit, Sangat sulit |
| ${interviewer}, do you have anything to add? | ${interviewer}, apakah ada tambahan? |  |  |

### Tanzanian Survey Instrument

Table A1.8. Survey instrument in English and Kiswahili

| **Question (English)** | **Question (Kiswhaili)** | **Response options (English)** | **Response options (Kiswahili)** |
| --- | --- | --- | --- |
| Section 1: Survey location (interviewer completes) | | | |
| Region | Region |  |  |
| District | District |  |  |
| What is the district? | What is the district? |  |  |
| Village | Village |  |  |
| Sub-village | Sub-village |  |  |
| Which is the nearest protected area? | Which is the nearest protected area? |  |  |
| Who is conducting the interview? | Who is conducting the interview? |  |  |
| What gender is the respondent? | What gender is the respondent? |  |  |
| Section 2: Experimental design details (not shown to respondent) | | | |
| Vignette order: ${v_order} | Vignette order: ${v_order} |  |  |
| Vignettes selected from block: ${block} | Vignettes selected from block: ${block} |  |  |
| Scenario number: ${rand_scenario} | Scenario number: ${rand_scenario} |  |  |
| Section 3: Consent | | | |
| My name is ${interviewer} and I work for a project being conducted by Bangor University in the United Kingdom. Today, I would like to invite you to take part in a short study.   The aim of the study is to understand what life is like for people like you that live close to a protected area. I am asking you to take part because you live close to a one. In total we are hoping to speak to between 200-300 people like you who live close to a protected area.   If you agree to take part, I will ask you some questions and record your answer on this device [show questionnaire on phone]. I will never record your name or your address on this, or any other form; your answers are private and confidential. The first set of questions is about you and your household, then I will ask about different activities you may or may not have done inside the protected area. Next, I will ask questions about your opinions and experiences of living in this area. This should take between 30 minutes and one hour.   Because I am not recording your name, your answers cannot be attributed to you, and it will not be possible to identify you from our records. Information you provide will not be shared with anybody outside of the research team and will only be used by members of the research team based in the UK. Your answers, together with those of 200-300 other people, will be used to write reports.   The study has been approved by the Bangor University Ethics Committee. If you would prefer not to take part, that is ok. Also, if you agree at first but then change your mind and would like to stop at any point, please tell me and I will stop immediately.   If you have any concerns please contact us on this phone number [provide contact card].   Before going any further, do you have any questions that you would like to ask me?   Would you like to take part in this study? | Jina langu naitwa ${interviewer} ni mtafiti msaidizi katika mradi unaotekelezwa na chuo kikuu cha Bangor kilichoko Uingereza. Ningependa kukukaribisha kushiriki katika utafiti huu mfupi.   Lengo la utafiti huu ni kuelewa juu ya maisha yakoje kwa watu kama nyinyi manoishi karibu na hifadhi. Nakuomba kushiriki kwasababu unaishi eneo hili ambalo lipo karibu na [Jina la hifadhi]. Kwa ujumla tunatarajia kuzungumza na wanakijiji 200-300 ambao wanaishi karibu na hifadhi.   Kama unakubali kushiriki nitakuuliza maswali machache na kujaza majibu yako kwenye kifaa hiki. Sitachukua jina lako au anwani yako na majibu yako yatakuwa ni siri. Kipengele cha kwanza cha maswali kinakuhusu wewe na kaya yako, halafu nitakuuliza kuhusu shughuli mbalimbali ambazo unawezakuwa umewahikufanya au hujawahi ndani ya hifadhi Baada ya hapo nitakuuliza maswali kuhusu maoni yako na uzoefu wako wa kuishi katika eneo hili. Mahojiano haya yatachukua muda wa dk45 mpaka saa moja.   Kwasababu sitaandika jina lako Majibu yako hayatahusianishwa na wewe na haitawezekana kukutambua kutoka kwenye majibu tuliyoandika. Taarifa utakazozitoa hazitatolewa kwa mtu yoyote nje ya timu hii ya utafiti na itatumika tu na watafiti wa timu hii walioko Uingereza. Majibu yako pamoja na wale watu wengine 200-300 yatatumika kuandika ripoti.   Utafiti huu umethibitishwa na Kamati ya Maadili ya Chuo kikuu cha Bangor. Kama hautapenda kushiriki katika mahojiano haya ni sawa, pia unaruhusiwa kuondoka kama utapata dharura au ukibadilisha mawazo wakati wowote tafadhali niambie na nitasitisha mahojiano mara moja.    Je, una swali lolote la kuniuliza kabla hatujaendelea?   Kama una wasiwasi wowote tafadhali wasiliana nasi kwa namba (mpe kadi ya mawasiliano)   Je, utapenda kushiriki katika utafiti huu? | Yes / No | Ndiyo / Hapana |
| • I confirm that I have had the ConHuB Information Sheet read out to me and I understand the information provided for this study.   • I have had the opportunity to ask questions if necessary and have had these answered satisfactorily.  • I understand that my participation is voluntary and that I am free to withdraw at any time without giving any reason. If I withdraw my data will be removed from the study and will be destroyed.  • I understand that the answers I provide will be used for the purposes detailed in the ConHuB Information Sheet.  Based upon the above, I agree to take part in this study | • Nakiri kwamba nimesoma/ kusomewa fomu ya [Conservation and Human Behavior-ConHuB] yaani Uhifadhi wa Mazingira na tabia za watu] na nimeelewa juu ya utafiti huu.   • Nilipewa nafasi ya kuuliza maswali na kupewa majibu yanayoridhisha.  • Naelewa kuwa kushiriki kwangu ni kwa hiari na niko huru kujitoa muda wowote nikipata dharura, na nikijitoa taarifa nilizotoa kwenye mjadala zitafutwa.   • Naelewa kwamba majibu niliyotoa yatatumika kwa kusudi lililotolewa kwenye fomu ya ConHuB ya ushiriki wa mahojiano   Kwa taarifa hapo juu nakubali kushiriki kwenye mjadala huu | Yes / No | Ndiyo / Hapana |
| Section 4: COVID-19 precautions | | | |
| While we understand that the government has announced that Tanzania has no COVID-19, because it is a dangerous disease, we are taking precautions to keep ourselves and everyone we meet safe in case there are cases that the government has not detected. | Wakati tunaelewa kwamba Serikali ya Tanzania imetangaza kutokuwa na ugonjwa wa COVID 19, kwasababu ni ugonjwa wa hatari , tunachukua tahadhari ili kuhakikisha usalama wetu na wa kila mmoja tunayekutana naye endapo kuna maambukizi ambayo serikali haijagundua bado. |  |  |
| Does anyone in your household have symptoms of COVID19 that have developed over the previous week?  These are:  • a new and persistent cough; • difficulty in breathing; • a high fever; • a recent loss of taste or smell? | Kuna mtu yeyote kwenye kaya yako ambaye ana dalili za COVID19 ambazo zimeonekana wiki iliyopita?   Hizi ni: • kikohozi kipya na endelevu; • kupumua kwa shida;  • homa kali;  • kupoteza uwezo wa kutambua harufu na ladha ya chakula hivi karibuni? | Yes / No | Ndiyo / Hapana |
| Thank the respondent for their time, explain that even though the sick individual may not have COVID we do not want to put other respondents at risk if they do have COVID. Wish them or their household member a quick recovery | Mshukuru muhojiwa kwa muda wake, mueleze kwamba ingawa mgonjwa anaweza akawa hauguwi COVID hatuhitaji kuweka washiriki wengine katika hatari kama watakuwa wanaugua COVID. Watakie wao au wanakaya/mwanakaya kupona haraka |  |  |
| Section 5: Respondent demographics | | | |
| I am going to ask you a few questions about yourself and the others in your household, by household I mean those who eat together and sleep in the same house, including children who may be at boarding school | Nitakuuliza mswali machache kuhusu wewe na watu wengine wa kaya yako, Ninaposema kaya ninamaanisha wale wote wanaokula pamoja na kulala kwenye nyumba moja ni kijuimuisha na watoto waliopo shule za bweni. |  |  |
| 1. How old are you? | 1. Una umri gani? |  |  |
| 2. What tribe do you belong to? | 2. Wewe ni kabila gani? |  |  |
| 2b. Please specify which tribe | 2b. Tafadhali taja kabila lako |  |  |
| 3. How many years have you lived in this village? | 3. Umeishi kwenye kijiji hiki kwa miaka mingapi? |  |  |
| 4. Are you currently attending school? | 4. Je, unasoma shule kwa sasa? |  |  |
| 5. How many years of schooling have you completed? | 5. Umesoma shule kwa miaka mingapi? |  |  |
| 6. How many other people live in this household? Both adults and children | 6. Watu wangapi wengine unaishi nao kwenye kaya yako? Watu wazima na watoto |  |  |
| 7. How many members of the household who are aged 13 years or older, have completed at least 7 years of schooling? | 7. Wanakaya wangapi ambao wana umri wa miaka 13 au zaidi, wamemaliza angalau miaka 7 ya shule? |  |  |
| 8. Are there any children in the household aged between 7 and 18 who do not go to school? | 8. Je, kwenye kaya kuna watoto ambao wako kati ya umri wa miaka 7 na 18 ambao hawaendi shule? |  |  |
| Section 6: Multi-dimensional measure of poverty | | | |
| 1. What is your households main livelihood activity? | 1. Je, ni shughuli gani kuu ya kujikimu katika kaya yako? |  |  |
| 2. Does the household's main residence have electricity? | 2. Je, kuna umeme au Solar kwenye makazi yenu ya kudumu? |  |  |
| 3. Does the household have access to a toilet? | 3. Je, kaya yako ina choo? |  |  |
| 3a. What type of toilet is this? | 3a. Taja ni aina gani ya choo |  |  |
| 3b. What is the 'other' type of toilet? | 3b. Taja aina 'nyingine' ya choo? |  |  |
| 4. Does the household share this toilet with other households? | 4. Je, choo cha kaya yako kinatumiwa na Kaya nyingine? |  |  |
| 5a. What is the household's main source of drinking water? | 5a. Chanzo kikuu cha maji ya kunywa kwenye kaya yako ni kipi? |  |  |
| 5b. What is the 'other' water source? | 5b. Taja chanzo 'kingine' cha maji ya kunywa kwenye kaya yako? |  |  |
| 6. How many minutes does it take to travel to the household's main source of drinking water? There and back using the mode of transport you use most often to collect water | 6. Unatumia dakika ngapi kwenda na kurudi kwenye chanzo kikuu cha maji kwa kutembea au kutumia usafiri unaoutumia mara kwa mara kwenda kufata maji? |  |  |
| 7a. What is the floor of the household's main residence made from? | 7a. Sakafu ya nyumba yenu ya kudumu imetengenezwa na nini? |  |  |
| 7b. What is the 'other' floor material? | 7b. Taja aina nyingine ya sakafu? |  |  |
| 8a. What is the household's main cooking fuel? | 8a. Taja chanzo kikuu cha nishati ya kupikia kwenye kaya yako? |  |  |
| 8b. What is the 'other' cooking fuel? | 8b. Taja chanzo 'kingine' cha nishati ya kupikia kwenye kaya yako? |  |  |
| 9. Has a child under the age of 18 died in the household in the last 5 years? | 9. Je, kwenye kaya yako kuna mtoto yeyote wa chini ya umri wa miaka 18 amefariki katika kipindi cha miaka 5 iliyopita? |  |  |
| 10. How many children under the age of 18 have died in the household in the last 5 years? | 10. Je, ni watoto wangapi wa chini ya umri wa miaka 18 wamefariki kwenye kaya yako katika kipindi cha miaka 5 iliyopita? |  |  |
| I am now going to ask about your households’ access to food over the last 12 months | Sasa, nitakuuliza maswali juu ya upatikanaji wa chakula kwenye kaya yako katika kipindi cha miezi 12 iliyopita. |  |  |
| 11. In the past 12 months, how often was there no food to eat of any kind in your house because of lack of resources to get food? | 11. Katika kipindi cha miezi 12 iliyopita, ni mara ngapi kumekosekana chakula cha aina yo yote, nyumbani kwako kutokana na ukosefu wa rasilimali ya kujipatia chakula? |  |  |
| 12. In the past 12 months, how often did any household member go to sleep at night hungry because there was not enough food? | 12. Katika kipindi cha miezi 12 iliyopita, ni mara ngapi mwanakaya yeyote amelala njaa usiku kwa sababu hakuna chakula cha kutosha? |  |  |
| 13. In the past 12 months, how often did any household member go a whole day and night without eating anything because there was not enough food? | 13. Katika kipindi cha miezi 12 iliyopita, ni mara ngapi mwanakaya yeyote kwenye kaya yako ameshinda mchana kutwa na kulala usiku kucha bila kula kitu chochote kwasababu hakuna chakula cha kutosha? |  |  |
| The following questions are about the items that you and your household own. | Maswali yafuatayo ni juu ya vitu unavyovimiliki wewe na kaya yako. |  |  |
| 14. How many radios does the household own? | 14. Kaya yako inamiliki radio ngapi? |  |  |
| 15. How many TVs does the household own? | 15. Kaya yako inamiliki runinga ngapi? |  |  |
| 16. How many mobile phones does the household own? | 16. Kaya yako inamiliki simu ngapi za mkononi? |  |  |
| 17. How many bicycles does the household own? | 17. Kaya yako inamiliki baiskeli ngapi? |  |  |
| 18. How many motorbikes does the household own? | 18. Kaya yako inamiliki Pikipiki ngapi? |  |  |
| 19. How many refrigerators does the household own? | 19. Kaya yako inamiliki majokofu mangapi? |  |  |
| 20. How many cars or trucks does the household own? | 20. Kaya yako inamiliki magari mangapi? |  |  |
| The next questions are about how you perceive yourself and your household. Please answer on a scale of strongly agree to strongly disagree | Maswali yafuatayo yanahusu jinsi unavyojichukulia wewe binafsi na kaya yako, tafadhali jibu kwa uwiano wa nakubali kabisa na kutokukubali kabisa |  |  |
| 21. Your household is poor | 21. Kaya yako ni masikini | Strongly agree, Agree, Neither agree or disagree, Disagree, Strongly disagree, Prefer not to answer, Don’t know | Nakubali kabisa,  Nakubali, Kutokufungamana, Kutokukubaliana,  Hukubaliani kabisa, Kupendelea kutokujibu,  Sijui |
| 22. Your household is happy | 22. Kaya yako inafuraha |  |  |
| 23. Yours is a prosperous household | 23. Kaya yako inamafanikio |  |  |
| 24. Community members are willing to help each other (e.g. with work, food or financially) | 24. Wanajamii wana utayari wa kusaidiana (mf. Kwenye kazi, chakula au kifedha) |  |  |
| 25. Trust among community members in this village is strong | 25. Uaminifu baina ya wanajamii katika kijiji hiki ni mkubwa |  |  |
| 26. Conflicts frequently arise between people or families in this community | 26. Migogoro baina ya watu na familia katika jamii hii inatokea mara kwa mara |  |  |
| 27. The amount of land your household has at the moment means you are prosperous | 27. Ukubwa wa ardhi unayomiliki kwa sasa unaonyesha mafanikio yako |  |  |
| 28. The amount of cattle you have at the moment means you are prosperous | 28. Idadi ya ng'ombe uliyonayo kwa sasa inaonyesha mafanikio yako |  |  |
| 29. How many acres do you think a family of five people needs for a good life? | 29. Unafikiri familia ya watu watano inahitaji kuwa na ekari ngapi ili kuwa na maisha mazuri? |  |  |
| 30. How many acres do you have? | 30. Je, wewe una ekari ngapi? |  |  |
| 31. How many cattle do you think a family of five people needs for a good life? | 31. Unafikiri familia ya watu watano inahitaji kuwa na ng'ombe wangapi ili kuwa na maisha mazuri? |  |  |
| 32. How many cattle do you have? | 32. Je, wewe unamiliki ng'ombe wangapi? |  |  |
| 33. ${interviewer}, any comments on the poverty section? | 33. ${interviewer}, any comments on the poverty section? |  |  |
| Section 7: Factorial Survey Experiment 🡪 ${v_X_q} – represents the vignette number shown to the respondent | | | |
| I will now read you several short descriptions of hypothetical interactions between rangers of the Protected Area and people who enter the National Park. For each one, I would like you to consider the situation, and tell me how fair your think the outcome is. | Sasa nitakusomea maelezo mafupi kadhaa ya mwingiliano wa kidhahania/kufikirika kati ya Askari wa wanyamapori wa Hifadhi na watu wanaoingia ndani ya Hifadhi. Kwa kila moja ningependa uzingatie hali hiyo na uniambie unafikiri matokeo ni ya haki kiasi gani. |  |  |
| Vignette 1 : ${v1_q} How fair do you think this? | Vignette 1 : ${v1_q} Unafikiri ni Haki kiasi gani? | Very fair, Fair, Neither fair nor unfair, Unfair, Very unfair, Prefer not to answer, Don’t know | Haki kabisa, Haki, Kutokufungamana, Sio Haki, Sio Haki kabisa, Kupendelea kutokujibu, Sijui |
| v1b. How likely do you think it is that this situation would happen in real life? | v1b. Unafikiri kuna uwezekano kiasi gani wa hali hii kutokea katika maisha halisi? | Very likely, Likely, Neither likely nor unlikely, Unlikely, Very unlikely, Prefer not to answer, Don’t know | Inawezekana kabisa, Inawezekana, Kutokufungamana, Haiwezekani, Haiwezekani kabisa, Kupendelea kutokujibu, Sijui |
| Vignette 2 : ${v2_q} How fair do you think this? | Vignette 2 : ${v2_q} Unafikiri ni Haki kiasi gani? | Very fair, Fair, Neither fair nor unfair, Unfair, Very unfair, Prefer not to answer, Don’t know | Haki kabisa, Haki, Kutokufungamana, Sio Haki, Sio Haki kabisa, Kupendelea kutokujibu, Sijui |
| v2b. How likely do you think it is that this situation would happen in real life? | v2b. Unafikiri kuna uwezekano kiasi gani wa hali hii kutokea katika maisha halisi? | Very likely, Likely, Neither likely nor unlikely, Unlikely, Very unlikely, Prefer not to answer, Don’t know | Inawezekana kabisa, Inawezekana, Kutokufungamana, Haiwezekani, Haiwezekani kabisa, Kupendelea kutokujibu, Sijui |
| Vignette 3 : ${v3_q} How fair do you think this? | Vignette 3 : ${v3_q} Unafikiri ni Haki kiasi gani? | Very fair, Fair, Neither fair nor unfair, Unfair, Very unfair, Prefer not to answer, Don’t know | Haki kabisa, Haki, Kutokufungamana, Sio Haki, Sio Haki kabisa, Kupendelea kutokujibu, Sijui |
| v3b. How likely do you think it is that this situation would happen in real life? | v3b. Unafikiri kuna uwezekano kiasi gani wa hali hii kutokea katika maisha halisi? | Very likely, Likely, Neither likely nor unlikely, Unlikely, Very unlikely, Prefer not to answer, Don’t know | Inawezekana kabisa, Inawezekana, Kutokufungamana, Haiwezekani, Haiwezekani kabisa, Kupendelea kutokujibu, Sijui |
| Vignette 4 : ${v4_q} How fair do you think this? | Vignette 4 : ${v4_q} Unafikiri ni Haki kiasi gani? | Very fair, Fair, Neither fair nor unfair, Unfair, Very unfair, Prefer not to answer, Don’t know | Haki kabisa, Haki, Kutokufungamana, Sio Haki, Sio Haki kabisa, Kupendelea kutokujibu, Sijui |
| v4b. How likely do you think it is that this situation would happen in real life? | v4b. Unafikiri kuna uwezekano kiasi gani wa hali hii kutokea katika maisha halisi? | Very likely, Likely, Neither likely nor unlikely, Unlikely, Very unlikely, Prefer not to answer, Don’t know | Inawezekana kabisa, Inawezekana, Kutokufungamana, Haiwezekani, Haiwezekani kabisa, Kupendelea kutokujibu, Sijui |
| Vignette 5 : ${v5_q} How fair do you think this? | Vignette 5 : ${v5_q} Unafikiri ni Haki kiasi gani? | Very fair, Fair, Neither fair nor unfair, Unfair, Very unfair, Prefer not to answer, Don’t know | Haki kabisa, Haki, Kutokufungamana, Sio Haki, Sio Haki kabisa, Kupendelea kutokujibu, Sijui |
| v5b. How likely do you think it is that this situation would happen in real life? | v5b. Unafikiri kuna uwezekano kiasi gani wa hali hii kutokea katika maisha halisi? | Very likely, Likely, Neither likely nor unlikely, Unlikely, Very unlikely, Prefer not to answer, Don’t know | Inawezekana kabisa, Inawezekana, Kutokufungamana, Haiwezekani, Haiwezekani kabisa, Kupendelea kutokujibu, Sijui |
| Vignette 6 : How fair do you think this?${v6_q} How fair do you think this? | Vignette 6 : ${v6_q} Unafikiri ni Haki kiasi gani? | Very fair, Fair, Neither fair nor unfair, Unfair, Very unfair, Prefer not to answer, Don’t know | Haki kabisa, Haki, Kutokufungamana, Sio Haki, Sio Haki kabisa, Kupendelea kutokujibu, Sijui |
| v6b. How likely do you think it is that this situation would happen in real life? | v6b. Unafikiri kuna uwezekano kiasi gani wa hali hii kutokea katika maisha halisi? | Very likely, Likely, Neither likely nor unlikely, Unlikely, Very unlikely, Prefer not to answer, Don’t know | Inawezekana kabisa, Inawezekana, Kutokufungamana, Haiwezekani, Haiwezekani kabisa, Kupendelea kutokujibu, Sijui |
| Vignette 7 : ${v7_q} How fair do you think this? | Vignette 7 : ${v7_q} Unafikiri ni Haki kiasi gani? | Very fair, Fair, Neither fair nor unfair, Unfair, Very unfair, Prefer not to answer, Don’t know | Haki kabisa, Haki, Kutokufungamana, Sio Haki, Sio Haki kabisa, Kupendelea kutokujibu, Sijui |
| v7b. How likely do you think it is that this situation would happen in real life? | v7b. Unafikiri kuna uwezekano kiasi gani wa hali hii kutokea katika maisha halisi? | Very likely, Likely, Neither likely nor unlikely, Unlikely, Very unlikely, Prefer not to answer, Don’t know | Inawezekana kabisa, Inawezekana, Kutokufungamana, Haiwezekani, Haiwezekani kabisa, Kupendelea kutokujibu, Sijui |
| Vignette 8 : ${v8_q} How fair do you think this? | Vignette 8 : ${v8_q} Unafikiri ni Haki kiasi gani? | Very fair, Fair, Neither fair nor unfair, Unfair, Very unfair, Prefer not to answer, Don’t know | Haki kabisa, Haki, Kutokufungamana, Sio Haki, Sio Haki kabisa, Kupendelea kutokujibu, Sijui |
| v8b. How likely do you think it is that this situation would happen in real life? | v8b. Unafikiri kuna uwezekano kiasi gani wa hali hii kutokea katika maisha halisi? | Very likely, Likely, Neither likely nor unlikely, Unlikely, Very unlikely, Prefer not to answer, Don’t know | Inawezekana kabisa, Inawezekana, Kutokufungamana, Haiwezekani, Haiwezekani kabisa, Kupendelea kutokujibu, Sijui |
| Section 8: Scenario | | | |
| Please tell me to what extent you agree or disagree with the following statements | Tafadhali niambie ni kwa kiwango gani unakubaliana au kutokukubaliana na sentensi zifuatazo |  |  |
| 1. Violating the Protected Area rules risks the good name of people in the community | 1. Kukiuka kanuni za hifadhi kuna hatarisha jina zuri la watu katika jamii | Strongly agree,  Agree,  Neither agree or disagree,  Disagree,  Strongly disagree,  Prefer not to answer,  Don’t know | Nakubali kabisa,  Nakubali,  Kutokufungamana,  Kutokukubaliana,  Hukubaliani kabisa,  Kupendelea kutokujibu,  Sijui |
| 2. The rules of the Protected Area support conservation of biodiversity | 2. Kanuni za Hifadhi zinasaidia uhifadhi wa bioanuai/viumbe hai |  |  |
| 3. The rules of the Protected Area are fair and consistent with the law | 3. Kanuni za Hifadhi ni haki na zinaendana na sheria |  |  |
| 4. Breaking rules is considered a disloyal towards fellow members of the community | 4. Kuvunja kanuni kunachukuliwa kama kutokuwa mwaminifu kwa wanajamii |  |  |
| I will now read you out an imaginary scenario. Based on this scenario I will ask you some questions, please answer them as honestly as you can. | Sasa nitakusomea mazingira ya kufikirika. Kulingana na mazingira nitakuuliza maswali, tafadhali jibu kwa ukweli uwezavyo. |  |  |
| Imagine the following situation. ${scenario} | Fikiria hali zifuatazo. ${scenario} |  |  |
| 5. If this scenario were true, how willing would you be, in general to follow the rules of the Protected Area? | 5. Ikiwa mazingira haya ni kweli, utakuwa tayari kiasi gani kufuata sheria za hifadhi kwa ujumla? | Very willing, Wiling, neither willing nor unwilling, Unwilling, Very unwilling, Prefer not to answer, Don’t know | Utayari kabisa, Utayari, Kutokufungamana, Kutokuwa na utayari, Kutokuwa na utayari kabisa, Kupendelea kutokujibu, Sijui |
| 6. If this scenario were true, how often in the coming months would you follow the rules of the Protected Area? | 6. Ikiwa mazingira haya ni kweli, ni mara ngapi utafuata sheria za hifadhi katika miezi ijayo? | Every Time, Almost every time, Occasionally, Rarely, Never, Prefer not to answer, Don’t know | Kila wakati, Karibu kila wakati, Mara kwa mara, Nadra, Kamwe, Kupendelea kutokujibu, Sijui |
| 7. If this scenario were true, to what extent would you consider violating the rules of the Protected Area? | 7. Ikiwa mazingira haya ni kweli, ni kwa kiwango gani unaweza kuamua kukiuka kanuni za hifadhi? | Definitely consider, May or may not consider, Would definitely not consider, Prefer not to answer, Don’t know | Kuamua bila shaka, Kuamua au kutokuamua, Kutokuamua, Kupendelea kutokujibu, Sijui |
| Section 9: End of survey | | | |
| The survey is now finished. Thank you for participating. | Utafiti sasa umeisha. Asante kwa kushiriki. |  |  |
| ${interviewer}, how engaged was the participant throughout the survey? | Je, muhojiwa alishiriki kiasi gani katika utafiti/ mahojiano? | Very engaged, Engaged, Neutral, Unengaged, Very unengaged | Kushiriki sana, Kushiriki, Kutokufungamana, Kutokushiriki, Kutokushiriki kabisa |
| ${interviewer}, how did you find surveying this individual? | Umeonaje/ilikuwaje kumuhoji mtu huyu? | Very easy, easy, Neutral, Hard, Very hard | Rahisi sana, Rahisi, Kutokufungamana, Ngumu, Ngumu sana |
| ${interviewer}, do you have anything to add? | Je, Unachochote cha kuongeza? |  |  |

## Appendix 2. Analyses

### Ordinal Regression Analyses

To assess the model fit of our ordinal regressions, we checked whether the proportional odd assumptions of models held by testing each model for nominal and scale effects (Christensen 2019b). However, we were advised that these tests were highly sensitive, and thus inappropriate for models that included hierarchical effects. Therefore, these checks were only run for the second experiment. Where identified as present, we included scale effects to relax the proportional odds assumptions for these variables and compared models that did and did not feature scale effects using a likelihood ratio test. We selected which model to use by determining whether there was a significant difference between the two models. We selected scale effects over nominal effects, as these offer greater flexibility for all values of predictor variables, and use fewer parameters, leading to more sensitive tests than including nominal effects (Christensen 2019b). Model results and the various processes undertaken are outlined below.

**Results table from experiment 1 (Fairness of sanctions):**

Table A2.1. Multi-level ordinal regression modelling the perceived fairness of different sanctions, where response is a 3-point Likert scale where (1= Unfair, 3=Fair). I260= crime asked about in Indonesia, T261= crime asked about in Tanzania. * = reference level, NP=National Park, PA=Protected Area

| ***Predictors*** | | ***Indonesia*** | | | ***Tanzania*** | | |
| --- | --- | --- | --- | --- | --- | --- | --- |
|  |  | ***Log-Odds*** | ***95% CIs*** | ***p*** | ***Log-Odds*** | ***95% CIs*** | ***p*** |
| Sanction administered | Arrest & prosecution | *Reference* | | | *Reference* | | |
|  | Warning & goods confiscated | 0.49 | -0.12 – 1.10 | 0.116 | -2.81 | -3.79 – -1.83 | **<0.001** |
|  | No sanction | -1.92 | -2.53 – -1.32 | **<0.001** | -7.15 | -8.45 – -5.85 | **<0.001** |
|  | Bribe & allowed to continue | -4.05 | -5.03 – -3.07 | **<0.001** | -8.28 | -9.92 – -6.64 | **<0.001** |
| Crime committed | Hunting | *Reference* | | | *Reference* | | |
|  | Logging | 0.28 | -0.18 – 0.73 | 0.237 | - | - | - |
|  | Grazing | - | - | - | -0.37 | -1.23 – 0.48 | 0.391 |
| Where offender is from | Someone from the community | *Reference* | | | *Reference* | | |
|  | Someone from outside the community | 0.41 | -0.05 – 0.87 | 0.081 | -0.18 | -1.02 – 0.66 | 0.677 |
| The power of the offender | Someone with little power | *Reference* | | | *Reference* | | |
|  | Someone with lots of power | 0.70 | 0.24 – 1.16 | **0.003** | 0.06 | -0.79 – 0.90 | 0.898 |
| ***Interactions*** | | | | | | | |
| Sanction X Behaviour | Warning X Logging / Grazing | 0.00 | -0.62 – 0.63 | 0.990 | 0.09 | -0.87 – 1.05 | 0.859 |
|  | No sanction X Logging / Grazing | -1.03 | -1.75 – -0.31 | **0.005** | 0.92 | -0.25 – 2.09 | 0.123 |
|  | Bribe X Logging / Grazing | -0.78 | -1.86 – 0.30 | 0.156 | 0.75 | -0.73 – 2.23 | 0.318 |
| Sanction X Actor | Warning X Outsider | -0.44 | -1.06 – 0.19 | 0.169 | 0.29 | -0.65 – 1.24 | 0.544 |
|  | No sanction X Outsider | -1.21 | -1.93 – -0.49 | **0.001** | 0.26 | -0.89 – 1.40 | 0.660 |
|  | Bribe X Outsider | -0.91 | -1.99 – 0.17 | 0.098 | 0.17 | -1.29 – 1.64 | 0.816 |
| Sanction X Power | Warning X Powerful person | -1.24 | -1.87 – -0.61 | **<0.001** | -0.20 | -1.16 – 0.75 | 0.680 |
|  | No sanction X Powerful person | -1.67 | -2.40 – -0.93 | **<0.001** | -0.08 | -1.24 – 1.07 | 0.886 |
|  | Bribe X Powerful person | -0.66 | -1.72 – 0.40 | 0.221 | 0.30 | -1.17 – 1.78 | 0.687 |
| ***Threshold coefficients*** | | | | | | | |
| Unfair (1) \| Neutral (2) | | -1.16 | -1.59 – -0.72 | **<0.001** | -3.69 | -4.60 – -2.78 | **<0.001** |
| Neutral (2) \| Fair (3) | | -0.65 | -1.08 – -0.23 | **0.003** | -3.59 | -4.50 – -2.68 | **<0.001** |
| ***Random effect*** | | | | | | | |
| *Respondent Id* | | *Var: 0.27 (SD: 0.52)* | | | *Var: 1.43 (SD: 1.20)* | | |
| N *(individuals)* | | 234 | | | 217 | | |
| Observations | | 1832 | | | 1734 | | |
| Log-Likelihood | | -952.85 | | | -578.50 | | |
| AIC | | 1941.70 | | | 1192.99 | | |
| Conditional number of Hessian | | 563.51 | | | 5567.60 | | |

### Analysis of data from Indonesia for Experiment 2

***Effect of corruption and descriptive norms on willingness to follow rules)***

Stage 1. A basic ordinal regression with all predictors (response = willingness to follow rules) was run on the Indonesian dataset.

summary(imod3)

## formula:

## intention3 ~ compliance + corruption + gender + age + education + MPI + attitude

## data: ind

##

## link threshold nobs logLik AIC niter max.grad cond.H

## logit flexible 221 -165.13 348.26 9(0) 3.21e-11 3.2e+05

##

## Coefficients:

## Estimate Std. Error z value Pr(>|z|)

## complianceLow -0.151673 0.319499 -0.475 0.6350

## corruptionHigh -2.106622 0.318377 -6.617 3.67e-11 ***

## gendermale 0.119660 0.330440 0.362 0.7173

## age 0.024106 0.018336 1.315 0.1886

## education 0.001232 0.051517 0.024 0.9809

## MPI -1.366007 2.189925 -0.624 0.5328

## attitude 0.476565 0.212829 2.239 0.0251 *

## ---

## Signif. codes: 0 '***' 0.001 '**' 0.01 '*' 0.05 '.' 0.1 ' ' 1

##

## Threshold coefficients:

## Estimate Std. Error z value

## Unwilling|Neither 0.2221 1.2500 0.178

## Neither|Willing 0.8744 1.2497 0.700

## (19 observations deleted due to missingness)

Stage 2. This model was then tested for nominal and scale effects. Results showed a significant scale effect for the variable ‘corruption’ (p=<0.05)

## Tests of nominal effects (imod3)

## formula: intention3 ~ compliance + corruption + gender + age + education + MPI + attitude

## Df logLik AIC LRT Pr(>Chi)

## <none> -165.13 348.26

## compliance 1 -165.07 350.13 0.12409 0.7246

## corruption 1 -164.41 348.82 1.44165 0.2299

## gender 1 -165.07 350.14 0.12063 0.7283

## age 1 -164.02 348.03 2.22374 0.1359

## education 1 -165.01 350.02 0.23704 0.6263

## MPI 1 -165.07 350.14 0.11858 0.7306

## attitude 1 -164.93 349.85 0.40467 0.5247

## Tests of scale effects (imod3)

## formula: intention3 ~ compliance + corruption + gender + age + education + MPI + attitude

## Df logLik AIC LRT Pr(>Chi)

## <none> -165.13 348.26

## compliance 1 -164.40 348.80 1.4567 0.22745

## corruption 1 -163.17 346.35 3.9103 0.04799 *

## gender 1 -165.08 350.16 0.0971 0.75529

## age 1 -165.10 350.20 0.0556 0.81357

## education 1 -164.72 349.44 0.8208 0.36495

## MPI 1 -164.62 349.24 1.0136 0.31404

## attitude 1 -164.96 349.92 0.3425 0.55836

## ---

## Signif. codes: 0 '***' 0.001 '**' 0.01 '*' 0.05 '.' 0.1 ' ' 1

Stage 3. The original model was then re-run, with the addition of a scale effect for ‘corruption’.

## formula:

## intention3 ~ compliance + corruption + gender + age + education + MPI + attitude

## scale: ~corruption

## data: ind

##

## link threshold nobs logLik AIC niter max.grad cond.H

## logit flexible 221 -163.17 346.35 13(0) 1.09e-11 3.6e+05

##

## Coefficients:

## Estimate Std. Error z value Pr(>|z|)

## complianceLow -0.06913 0.43910 -0.157 0.8749

## corruptionHigh -2.49301 0.54096 -4.608 4.06e-06 ***

## gendermale 0.23221 0.45840 0.507 0.6125

## age 0.04725 0.02730 1.731 0.0835 .

## education 0.03606 0.07264 0.496 0.6196

## MPI -0.37161 3.25330 -0.114 0.9091

## attitude 0.75885 0.30771 2.466 0.0137 *

## ---

## Signif. codes: 0 '***' 0.001 '**' 0.01 '*' 0.05 '.' 0.1 ' ' 1

##

## log-scale coefficients:

## Estimate Std. Error z value Pr(>|z|)

## corruptionHigh 0.7459 0.3641 2.048 0.0405 *

## ---

## Signif. codes: 0 '***' 0.001 '**' 0.01 '*' 0.05 '.' 0.1 ' ' 1

##

## Threshold coefficients:

## Estimate Std. Error z value

## Unwilling|Neither 2.134 1.864 1.145

## Neither|Willing 3.155 1.956 1.613

## (19 observations deleted due to missingness)

Stage 4. Both models were compared using a Likelihood Ratio test. Results showed that the model that included a Scale effect for ‘corruption’ performed better (p=<0.05). This model is presented in the results.

anova(imod3, imod4)

## Likelihood ratio tests of cumulative link models:

## formula:

## imod3 intention3 ~ compliance + corruption + gender + age + education + MPI + attitude

## imod4 intention3 ~ compliance + corruption + gender + age + education + MPI + attitude

## scale: link: threshold:

## imod3 ~1 logit flexible

## imod4 ~corruption logit flexible

##

## no.par AIC logLik LR.stat df Pr(>Chisq)

## imod3 9 348.26 -165.13

## imod4 10 346.35 -163.17 3.9103 1 0.04799 *

## ---

## Signif. codes: 0 '***' 0.001 '**' 0.01 '*' 0.05 '.' 0.1 ' ' 1

### Analysis of data Tanzania for Experiment 2

***(Effect of corruption and descriptive norms on willingness to follow rules)***

Stage 1. A basic ordinal regression with all predictors (response = willingness to follow rules) was run on the Tanzanian dataset.

summary(tmod3)

## formula:

## intention3 ~ compliance + corruption + gender + age + education + MPI + attitude

## data: tz

##

## link threshold nobs logLik AIC niter max.grad cond.H

## logit flexible 211 -93.68 205.36 8(2) 1.79e-13 2.3e+05

##

## Coefficients:

## Estimate Std. Error z value Pr(>|z|)

## complianceLow -0.136399 0.407424 -0.335 0.73779

## corruptionHigh 0.415785 0.411822 1.010 0.31268

## gendermale -1.732575 0.471799 -3.672 0.00024 ***

## age 0.028058 0.018132 1.547 0.12176

## education -0.004424 0.083192 -0.053 0.95759

## MPI -3.659562 1.920188 -1.906 0.05667 .

## attitude 0.641299 0.248995 2.576 0.01001 *

## ---

## Signif. codes: 0 '***' 0.001 '**' 0.01 '*' 0.05 '.' 0.1 ' ' 1

##

## Threshold coefficients:

## Estimate Std. Error z value

## Unwilling|Neither 0.1743 1.6486 0.106

## Neither|Willing 0.3886 1.6487 0.236

## (13 observations deleted due to missingness)

Stage 2. This model was then tested for nominal and scale effects. Results showed a significant scale effect for the variable

‘compliance’ (p=<0.01) and negligible effects for corruption and attitude

## Tests of nominal effects(tmod3)

## formula: intention3 ~ compliance + corruption + gender + age + education + MPI + attitude

## Df logLik AIC LRT Pr(>Chi)

## <none> -93.681 205.36

## compliance 1 -92.380 204.76 2.6019 0.106733

## corruption 1 -93.675 207.35 0.0117 0.913713

## gender 1 -93.352 206.70 0.6570 0.417630

## age

## education 1 -89.990 199.98 7.3806 0.006593 **

## MPI

## attitude 1 -83.357 186.71 20.6479 5.52e-06 ***

## ---

## Signif. codes: 0 '***' 0.001 '**' 0.01 '*' 0.05 '.' 0.1 ' ' 1

## Tests of scale effects (tmod3)

## formula: intention3 ~ compliance + corruption + gender + age + education + MPI + attitude

## Df logLik AIC LRT Pr(>Chi)

## <none> -93.681 205.36

## compliance 1 -89.969 199.94 7.4236 0.006437 **

## corruption 1 -92.002 204.00 3.3578 0.066887 .

## gender 1 -93.613 207.23 0.1347 0.713572

## age 1 -93.666 207.33 0.0289 0.864951

## education 1 -93.678 207.35 0.0058 0.939053

## MPI 1 -93.185 206.37 0.9914 0.319403

## attitude 1 -92.157 204.31 3.0468 0.080895 .

## ---

## Signif. codes: 0 '***' 0.001 '**' 0.01 '*' 0.05 '.' 0.1 ' ' 1

Stage 3. The original model was then re-run, with the addition of a scale effect for ‘compliance’.

summary(tmod4)

## formula:

## intention3 ~ compliance + corruption + gender + age + education + MPI + attitude

## scale: ~compliance

## data: tz

##

## link threshold nobs logLik AIC niter max.grad cond.H

## logit flexible 211 -89.97 199.94 13(4) 9.04e-07 2.8e+05

##

## Coefficients:

## Estimate Std. Error z value Pr(>|z|)

## complianceLow -0.97357 0.35786 -2.721 0.00652 **

## corruptionHigh 0.27445 0.21362 1.285 0.19888

## gendermale -1.03421 0.41000 -2.522 0.01165 *

## age 0.02509 0.01158 2.166 0.03032 *

## education 0.02646 0.03684 0.718 0.47265

## MPI -1.73978 1.08230 -1.607 0.10795

## attitude 0.38009 0.17381 2.187 0.02876 *

## ---

## Signif. codes: 0 '***' 0.001 '**' 0.01 '*' 0.05 '.' 0.1 ' ' 1

##

## log-scale coefficients:

## Estimate Std. Error z value Pr(>|z|)

## complianceLow -1.1204 0.4337 -2.583 0.00979 **

## ---

## Signif. codes: 0 '***' 0.001 '**' 0.01 '*' 0.05 '.' 0.1 ' ' 1

##

## Threshold coefficients:

## Estimate Std. Error z value

## Unwilling|Neither 0.1200 0.9301 0.129

## Neither|Willing 0.2390 0.9451 0.253

## (13 observations deleted due to missingness)

Stage 4. Both models were compared using a Likelihood Ratio test. Results showed that the model that included a Scale effect for ‘compliance’ performed better (p=<0.01). This model is presented in the results.

anova(tmod3, tmod4)

## Likelihood ratio tests of cumulative link models:

##

## formula:

## tmod3 intention3 ~ compliance + corruption + gender + age + education + MPI + attitude

## tmod4 intention3 ~ compliance + corruption + gender + age + education + MPI + attitude

## scale: link: threshold:

## tmod3 ~1 logit flexible

## tmod4 ~compliance logit flexible

##

## no.par AIC logLik LR.stat df Pr(>Chisq)

## tmod3 9 205.36 -93.681

## tmod4 10 199.94 -89.969 7.4236 1 0.006437 **

## ---

## Signif. codes: 0 '***' 0.001 '**' 0.01 '*' 0.05 '.' 0.1 ' ' 1
